# MambaIR-YOLO: A Feature-Guided Lightweight State-Space Framework for Aerial Small-Object Detection

**DOI:** 10.3390/s26144517

**Published:** 2026-07-16

**Authors:** Hongsen Rao, Lin Tian, Nan Li, Xinyue Luo

**Affiliations:** 1 School of Electronic Engineering, Yili Normal University, Yining 835000, China; 20250050211@ylnu.edu.cn (H.R.); 20240050186@ylnu.edu.cn (N.L.); 20250050212@ylnu.edu.cn (X.L.); 2 Yili Key Laboratory of Intelligent Computing Research and Application, Yili Normal University, Yining 835000, China

**Keywords:** aerial remote sensing, small-object detection, YOLOv5, state-space model, mamba, super-resolution guidance, lightweight network

## Abstract

To address the challenges of extremely small object scales, weak texture information, and severe background interference in aerial remote sensing images, we propose MambaIR-YOLO, a feature-guided lightweight state-space framework for aerial small-object detection. Based on the YOLOv5 architecture, this method introduces systematic improvements in three key areas—fine-grained feature modeling, long-range dependency learning, and high-resolution spatial information preservation—while ensuring real-time performance. Specifically, a feature-level MambaIR_SR (feature-level Mamba-based super-resolution guidance) training auxiliary branch is designed to generate high-resolution detail-guided information by fusing shallow-level detail features with deep-level semantic features, improving the representation of small-object edges and textures during the training phase. In the main network, we introduce the Object Detail State-Space Block (ODSSBlock), driven by Lightweight Mamba. Through a channel-compressed state-space modeling mechanism, the ODSSBlock unifies local detail preservation and long-range context modeling with low computational overhead. Concurrently, a Feature Modulation Block (FMB) is constructed at the shallow feature level to enhance the representation of high-frequency structural information, thereby mitigating the irreversible detail degradation caused by multiple downsampling steps. In the feature fusion and detection stages, we introduce a Coordinate and Channel Attention (C3CA) attention enhancement module and construct a lightweight decoupled detection head based on a Lightweight Decoupled Head (LADH). This performs single-scale dense prediction on high-resolution features, improving small-object localization and reducing information loss. Notably, the MambaIR_SR branch is exclusively utilized for optimization during the training phase and is entirely discarded during inference, introducing no additional computational overhead. Experimental results on the VEDAI dataset demonstrate that the proposed method achieves an average mAP50 of 84.19 ± 0.03% over three independent runs, with only 4.46 M parameters and 19.97 GFLOPs, outperforming the baseline methods while maintaining low computational cost.

## 1. Introduction

The rapid development of drone and aerial remote sensing imaging technologies has made it possible to observe ground targets over large areas in a timely manner. Target detection in aerial remote sensing imagery has been widely applied to tasks such as intelligent traffic monitoring [1], urban management [2], disaster relief [3], military reconnaissance [4], agricultural field inspections [5], and public safety. Compared to natural scene imagery, the accurate detection of targets in aerial remote sensing images presents unique challenges. Consequently, accurate detection of these targets is a key research problem in remote sensing.

However, object detection in aerial remote sensing images still faces significant challenges. First, due to the high altitude at which images are captured, typical objects such as vehicles often occupy only a small number of pixels in the image, making them prone to low resolution [1,2], weak texture [4], and blurred edges [6]. Second, the background in aerial images is typically very complex; road textures, building edges, shadows, vegetation, and noisy areas may exhibit visual responses similar to those of small objects, leading to false positives in the model [3,4,6]. Furthermore, aerial images feature a wide field of view and a complex distribution of scales; a single image may simultaneously contain both large-scale background structures and extremely small targets, requiring detection networks to possess both fine-grained local perception capabilities [7] and strong contextual modeling capabilities [8]. Finally, in drone inspection and real-time edge monitoring scenarios, detection models must also meet deployment requirements for low parameter counts, low computational demands [2], and high inference speeds [9,10].

In recent years, the YOLO series of detectors has been widely used in real-time object detection tasks due to its high inference speed and good detection accuracy [11,12,13]. YOLOv5 achieves a balance between speed and accuracy through a convolutional backbone and pyramid-based multi-scale feature fusion [12,14]. However, detecting extremely small aerial targets remains challenging for conventional YOLO-based detectors [4]. This is partly because conventional convolutions primarily model local neighborhoods, while repeated downsampling and low-resolution feature maps may attenuate the already weak responses of small objects [7,8].

To address the issue of feature degradation in small-object detection in aerial imagery, previous studies have attempted to incorporate methods such as super-resolution enhancement, multi-scale feature fusion [7], attention mechanisms [15,16,17], and global context modeling [8,18]. Image-level SR-assisted detection has been used to improve the visual details of small objects [19]. Other studies further integrate SR branches with detection networks to enhance edge and texture representation [20,21]. Attention mechanisms can highlight target-relevant regions while suppressing interference from complex backgrounds [17]. Global modeling architectures such as Transformer and Mamba enhance the model’s ability to represent long-range dependencies. Among these, Mamba—a sequence modeling method based on a state-space model—can model long-range dependencies [22] with low computational complexity, offering new insights for lightweight visual modeling [23,24]. However, existing methods still have some shortcomings: First, some methods treat the super-resolution module as a branch in the inference stage [8], leading to increased computational complexity [19,20]. Second, some Mamba-based visual and detection architectures primarily emphasize efficient global modeling, whereas explicit preservation of shallow details for extremely small targets remains comparatively underexplored [25,26,27]. Third, although multi-scale fusion enhances cross-level feature interaction, preserving high-resolution localization information for extremely small targets remains challenging [14,28].

A review of existing research reveals that three key challenges in small-target detection using aerial remote sensing remain unresolved. First, small targets account for an extremely small proportion of pixels in the input images, with limited edge, texture, and structural information; after successive downsampling, irreversible detail degradation is likely to occur [28]. Second, while convolutional networks excel at local modeling, their ability to capture long-range contextual information is limited; however, directly introducing complex global modeling modules may compromise the real-time performance of lightweight detectors [29,30]. Third, in conventional multi-scale detection architectures, deep low-resolution detection layers are not always effective for localizing small targets [28]. Therefore, how to leverage deep semantic information while preserving high-resolution spatial information remains a critical challenge in aerial small-target detection.

We propose MambaIR-YOLO, a feature-guided lightweight state-space detection framework for aerial small-object detection. The proposed framework integrates training-time feature-level detail guidance, lightweight state-space modeling, and high-resolution single-scale prediction to improve small-object detection while maintaining low computational cost. Based on the YOLOv5 framework, the model is designed around three key aspects: “feature-level detail guidance, lightweight state-space modeling, and high-resolution single-scale detection.” First, we construct a feature-level MambaIR_SR training auxiliary branch that utilizes shallow detail features and deep semantic features from the main network to generate high-resolution detail guidance information, enhancing the learning of small-target edges, textures, and structural features during the training phase. Second, we design a Lightweight Mamba-driven ODSSBlock, introducing a channel-compressed state-space modeling unit within a C3-like branch structure to enhance local detail preservation and long-range context representation capabilities at a lower computational cost. Concurrently, we introduce a Feature Modulation Block (FMB) into the shallow layers of the backbone to reinforce high-frequency details in early features, mitigating the feature degradation of small objects before and after continuous downsampling. Finally, we abandon the conventional Feature Pyramid Network–Path Aggregation Network (FPN-PAN) multi-scale detection output of YOLOv5, retaining only the top-down Feature Pyramid Network (FPN) semantic propagation path. A C3CA attention module is introduced at the fusion node, and dense prediction is performed on the high-resolution single-scale feature layer, better preserving the spatial position information of small aerial targets.

The core idea of the method presented in this paper is that the key to detecting small targets in aerial remote sensing lies not merely in increasing network depth or stacking complex feature fusion structures but in simultaneously addressing three challenges—“early-stage detail degradation,” “insufficient long-range context,” and “preservation of high-resolution localization information”—while adhering to lightweight constraints. Unlike traditional image-level super-resolution-assisted detection methods, the MambaIR_SR branch in this paper is primarily used for feature-level detail guidance during the training phase and does not participate in the final detection computation during the inference phase; therefore, it does not increase the number of parameters or GFLOPs of the final deployed model. Unlike existing detection methods that directly stack Mamba modules, we reduce state-space modeling overhead through the channel bottleneck design of Lightweight Mamba and embed it separately into ODSSBlock and FMB to balance local detail preservation, long-range context modeling, and lightweight inference. Unlike conventional multi-scale detection architectures, this paper focuses more on the role of high-resolution fused features in locating extremely small aerial targets, using a single-scale high-resolution detection head to mitigate the adverse effects of low-resolution detection layers on small-target localization.

Systematic experiments were conducted on the VEDAI aerial remote sensing dataset. The proposed method achieved an average mAP50 of 84.19 ± 0.03% over three independent runs, with only 4.4588 million parameters and a computational cost of 19.97 GFLOPs. Compared with recent YOLO-series detectors such as YOLOv8, YOLOv11, and YOLO26, the proposed method achieves higher mAP50 and mAP50–95 while maintaining a lightweight model size and competitive inference speed. It also outperforms SuperYOLO in both mAP50 and mAP50–95, demonstrating a favorable balance between small-object detection accuracy and deployment efficiency.

Our main contributions are fourfold:

1. We propose a lightweight, high-resolution, single-scale MambaIR-YOLO framework for small-object detection in aerial remote sensing. Building upon YOLOv5, this framework combines feature-level detail guidance during training, lightweight state-space modeling, and a high-resolution single-scale prediction strategy to enhance small-object detection performance while maintaining a low number of parameters and computational cost.
2. We construct a feature-level MambaIR_SR training auxiliary branch. This branch utilizes shallow detail features and deep semantic features from the main network to generate high-resolution detail guidance information, which is used to enhance the detection network’s learning of small-object edges, textures, and structural information; during inference, this branch is removed, thus it does not increase the parameter count or GFLOPs of the final deployed model.
3. We design a Lightweight Mamba-driven ODSSBlock and a shallow FMB. The ODSSBlock embeds channel-compressed Mamba units within a C3-like branch structure to balance local detail preservation with long-range context modeling; the FMB is deployed in the shallow layers of the backbone, where it enhances early high-frequency features through depthwise convolutions and lightweight Mamba modeling, thereby mitigating the degradation of small-object details before and after downsampling.
4. We construct the C3CA-LADH high-resolution single-scale detection head and introduce a CIoU-NWD hybrid localization loss. C3CA is used to enhance key channel and spatial position responses in the fused features, while LADH decouples the branches to perform bounding box regression, target confidence prediction, and class prediction, respectively; the CIoU-NWD hybrid localization loss further improves the model’s robustness for bounding box regression of small aerial targets.

## 2. Related Work

### 2.1. Small-Object Detection in Aerial Remote Sensing

Target detection in aerial remote sensing imagery is a critical task in intelligent remote sensing interpretation and UAV visual perception, with widespread applications in traffic monitoring [1], urban management, disaster relief, military reconnaissance, agricultural field inspections, and public safety [5]. Compared to natural scene imagery, aerial remote sensing images are typically captured by UAVs or high-altitude platforms and are characterized by a wide field of view, a bird’s-eye-view perspective, complex background information, and significant variations in target scale [1,4]. In such images, ground targets such as vehicles often occupy only a small area of the image, with a limited number of target pixels and weak edge and texture features [6]. Therefore, small-object detection in aerial images requires highly accurate recognition and localization capabilities [3,4].

In recent years, the YOLO series of detectors has been widely applied to aerial remote sensing target detection tasks due to their high inference efficiency and good detection accuracy [2,13]. Single-stage detectors such as YOLOv5 and YOLOv7 achieve fast detection through end-to-end prediction and multi-scale feature maps [12,13]. YOLO-based methods generally provide favorable inference efficiency, making them suitable for real-time applications [31,32]. However, the detection of small targets in aerial remote sensing differs significantly from conventional object detection. Small targets inherently contain limited information in the input image, and after multiple rounds of downsampling, their spatial positions and edge textures may be further degraded, leading to false negatives and localization errors in complex backgrounds [4,6].

To address the challenges of detecting small targets in aerial images, existing research has explored approaches such as improving the backbone network [4], enhancing neck feature fusion [7,14], introducing attention mechanisms, and optimizing detection heads [28]. Some methods introduce high-resolution shallow features into the detection stage by adding shallow detection heads or improving the feature pyramid structure, thereby mitigating the issue of insufficient responses for small targets in deep, low-resolution feature maps [28]. Other methods enhance the discriminative capability of target regions in complex scenes by incorporating context modeling modules, attention mechanisms [17], or multimodal information fusion [19]. While these methods have improved the performance of small-object detection in aerial remote sensing to some extent, issues such as increased parameter counts, higher inference complexity, and inadequate handling of detail degradation in shallow layers persist [20].

Recently, researchers have proposed various customized YOLO architectures and multi-scale fusion networks specifically tailored for aerial small-object detection. For example, UAV-YOLOv8 [31] introduced an improved YOLOv8 model optimized for UAV aerial photography scenarios to enhance small-object perception. Similarly, MFFSODNet [32] designed a multiscale feature fusion network for aerial images to address the severe feature degradation of tiny objects. While these specialized methods have made significant progress, the detection of extremely small targets with weak textures in complex backgrounds still requires further structural innovation to balance fine-grained feature preservation and long-range context modeling without compromising inference speed.

Therefore, small-object detection in aerial remote sensing still requires a model architecture that balances detection accuracy, detail preservation, and inference efficiency. The MambaIR-YOLO proposed in this paper is designed precisely to address this need. Unlike traditional YOLO improvements, this method does not simply increase the depth or complexity of the detection network. Instead, it constructs a unified framework centered on detail-guided learning, lightweight state-space modeling, and high-resolution single-scale detection: on one hand, it utilizes YOLOv5 to maintain the real-time performance of a single-stage detector; on the other hand, it introduces the MambaIR_SR training auxiliary branch to enhance the learning of small-target details, while combining ODSSBlock, FMB, and C3CA to improve local–global features and target response capabilities in complex backgrounds.

### 2.2. Super-Resolution-Assisted Object Detection

The performance of small-object detection is significantly affected by object resolution [21]. In aerial remote sensing imagery, objects are typically captured from a bird’s-eye view by high-altitude platforms or drones, and objects such as vehicles often occupy only a limited number of pixels in the raw image [6]. When the target area is too small or the image quality is low, the edge, texture, corner, and shape information of the target becomes very faint, making it difficult for detection models to distinguish real targets from clutter in complex backgrounds [20]. Particularly in deep detection networks, after the input image undergoes multiple convolutions and downsampling, the already limited high-frequency details of small targets are further attenuated, ultimately leading to unstable feature responses, inaccurate localization, and increased false negative rates [21].

Super-resolution reconstruction offers an effective approach to mitigate these issues. Image super-resolution aims to recover higher-resolution visual representations from low-resolution images, enhancing the recognizability of weak targets by amplifying edge, texture, and structural details [33,34]. Unlike methods that rely solely on enhancement within the detection network, super-resolution-assisted detection improves the representation of target information at the input stage or during learning, enabling the detection network to access richer, finer-grained information [20]. For small targets in aerial remote sensing, this strategy is particularly effective, as one of the root causes of detection failure for small targets is precisely the insufficient representation of target details [21].

In recent years, some studies have attempted to combine super-resolution with object detection to improve the performance of small-target detection. Relevant methods typically achieve this by constructing super-resolution branches, image enhancement modules, or high-resolution auxiliary inputs, enabling the detection network to obtain clearer information about target structures [35]. These methods demonstrate that super-resolution not only improves image visual quality but also provides additional detail supervision for detection models [21]. However, existing super-resolution-assisted detection methods still have certain limitations. First, many methods treat the super-resolution network as an explicit inference branch within the detection model; while this can improve detection accuracy, it also increases model parameters, computational complexity, and inference latency [20]. Second, image-level super-resolution typically optimizes for visual reconstruction quality; its output is not necessarily optimal for the detection task and may even introduce spurious texture artifacts in complex backgrounds [34]. Finally, when there is a lack of effective coupling between the super-resolution branch and the detection task, optimization inconsistencies may arise between the reconstruction objective and the detection objective [35].

Based on the above analysis, this paper extends the concept of super-resolution assistance from image-level reconstruction to feature-level detail guidance. In MambaIR-YOLO, the MambaIR_SR branch does not perform super-resolution reconstruction directly on the original input image. Instead, it utilizes shallow detail features and deep semantic features from the main network for alignment, stitching, and reconstruction, guiding the model to learn more discriminative high-resolution detail representations of small objects during training. During the inference stage, this auxiliary branch is removed, thus avoiding any increase in the final detection model’s parameter count or computational load. This design ensures that super-resolution assistance no longer serves merely visual reconstruction but directly addresses the learning process within the detection network, better meeting the dual requirements of fine-grained representation and real-time deployment in small-object detection for aerial remote sensing.

### 2.3. State-Space Models and Mamba Visual Modeling

Convolutional neural networks (CNNs) have long served as the core backbone output architecture for object detection tasks. CNNs extract low-level visual features such as edges, textures, and shapes through local convolutional kernels and gradually achieve larger receptive fields and stronger semantic representation capabilities through stacked layers [12,13]. For conventional object detection tasks, convolutional architectures offer advantages such as parameter sharing, computational efficiency, and strong local modeling capabilities and are therefore widely used in detection frameworks such as YOLO, Faster R-CNN, and RetinaNet. However, relying solely on convolutional structures still has certain limitations in small-object detection tasks in aerial remote sensing. Due to the vast scene scope and complex background structures of aerial images, small objects have close contextual relationships with surrounding roads, buildings, and shadowed areas; models relying solely on local convolutions may struggle to adequately model these long-range dependencies [8,18].

To enhance global context modeling capabilities, the Transformer has been introduced into computer vision tasks [33]. Self-attention models global dependencies between image regions [36]. For object detection, global context helps improve discrimination capabilities in scenarios involving occluded objects, dense objects, and complex backgrounds [33]. However, self-attention computations in Transformers are typically computationally intensive [30]. Particularly in high-resolution aerial remote sensing images, where input feature sequences are long, computational costs and memory usage increase significantly [29,30]. Consequently, while Transformer architectures possess strong global modeling capabilities, they are not always suitable for lightweight real-time detection models.

In recent years, state-space models and their representative architecture, Mamba, have provided new insights for modeling long-range dependencies in computer vision [22,23]. Unlike Transformers, which rely on global self-attention, Mamba models sequence information through a selective state-space mechanism, enabling efficient long-range dependency modeling with linear complexity. This achieves a good balance between efficiency and global modeling capability. As Mamba has been extended to tasks such as image classification, image restoration, object detection, and remote sensing image processing, researchers have begun to apply it to address the shortcomings of convolutional networks in modeling long-range context [25,27]. Existing Mamba-YOLO-style methods demonstrate that introducing state-space models into detection frameworks helps enhance the ability to represent the relationship between target regions and their context in complex backgrounds [25].

Although Mamba shows strong potential in visual detection tasks, existing Mamba-YOLO-style methods still have certain shortcomings. First, existing visual Mamba methods primarily emphasize efficient global representation learning, whereas explicit preservation of shallow local details for extremely small objects remains less explored [25,27]. For small targets in aerial remote sensing, objects typically consist of only a few pixels, and class discrimination and localization rely heavily on limited local details. If a model overemphasizes global context while failing to preserve local structures, the features of small targets may be overshadowed by background semantics. Second, directly stacking Mamba modules on full-channel features may still introduce additional parameters and computational complexity, hindering lightweight deployment [25].

Based on the above analysis, this paper does not simply treat Mamba as a stacked module within the backbone network. Instead, addressing the trade-off between local detail preservation and global context modeling in small-target detection, we design the Lightweight Mamba unit and integrate it into ODSSBlock and FMB. Lightweight Mamba first reduces the channel dimension via a 1 × 1 convolution to perform state-space modeling in a low-dimensional feature space and then restores the channel representation through another 1 × 1 convolution, while incorporating residual connections to maintain feature stability. This design enables efficient long-range modeling while preserving small-object details.

### 2.4. Attention Mechanisms and Lightweight Enhancement

In complex remote sensing scenarios, small targets are often intermixed with background areas such as roads, buildings, vegetation, and shadows, resulting in weak target responses and strong background interference. Attention mechanisms enhance responses in key regions and suppress irrelevant background information by adaptively weighting channel, spatial, or positional information and are therefore widely used to improve object detection models [16]. Common attention mechanisms include channel attention, spatial attention, hybrid attention, and coordinate attention [17]. Among these, coordinate attention encodes horizontal and vertical positional information separately, preserving spatial positional information while enhancing channel responses, which offers certain advantages for small-object localization [17].

In the YOLO series of detectors, researchers often embed attention mechanisms into the backbone network, feature fusion layers, or detection heads to enhance target-relevant features [17]. For small-object detection in remote sensing, attention mechanisms help models highlight target regions within complex backgrounds, demonstrating particular effectiveness in scenarios involving dense vehicle clusters, low-contrast targets, and occlusion [17]. However, attention mechanisms alone cannot fully resolve the issue of small-object features gradually degrading during early downsampling [7]. If shallow-layer high-frequency details are already weakened before entering the fusion stage, simply adding attention modules in the neck or detection heads remains insufficient to fully recover the object’s edge and structural information.

Therefore, lightweight enhancement must address two levels simultaneously: first, reinforcing small-object details as early as possible in the shallow layers of the backbone to prevent premature loss of high-frequency information before successive downsampling; second, enhancing the response to target regions during the feature fusion stage to reduce interference from complex backgrounds. This paper designs an FMB feature modulation module in the shallow layers of the backbone, which enhances local edge and texture features through depthwise convolutions and utilizes Lightweight Mamba for lightweight context interaction, improving the resolvability of small-object features before they enter deep downsampling. Concurrently, this paper introduces a C3CA module at the Neck fusion node, combining a coordinate attention mechanism with a C3-like structure to enhance target-relevant channel and spatial position responses in the fused features. It should be noted that this paper does not treat the attention mechanism itself as the primary innovation, but rather as a fusion enhancement component within a high-resolution single-scale detection framework, working in conjunction with FMB, ODSSBlock, and MambaIR_SR to support the preservation of small-target details and the learning of discriminative features in aerial remote sensing.

In summary, although existing methods have made some progress in multi-scale fusion, super-resolution assistance, attention enhancement, and global context modeling, issues such as insufficient feature-level detail guidance, limited lightweight long-range modeling capabilities, and inadequate preservation of high-resolution localization information still persist in the detection of small targets in aerial remote sensing. To address these shortcomings, we propose improvements in three aspects: feature-level training assistance for MambaIR_SR, Lightweight Mamba-driven ODSSBlock and FMB, and high-resolution single-scale detection.

## 3. Methodology

### 3.1. Overall Network Architecture

To address issues such as low target resolution, degradation of shallow-level details, insufficient modeling of long-range context, and loss of high-resolution localization information in the detection of small targets in aerial remote sensing, we propose a MambaIR-guided, lightweight, high-resolution, single-scale YOLO detection model named MambaIR-YOLO tailored for aerial small-target detection in remote sensing. Based on the YOLOv5 [12] detection framework, this model retains the efficient inference capabilities of a single-stage detector while introducing a feature-level MambaIR_SR training auxiliary branch, an ODSSBlock driven by Lightweight Mamba [22], an FMB shallow feature modulation module, a C3 module with a C3CA fusion enhancement module, and a high-resolution single-scale detection head. This results in a lightweight single-scale detection framework.

**Figure 1:**
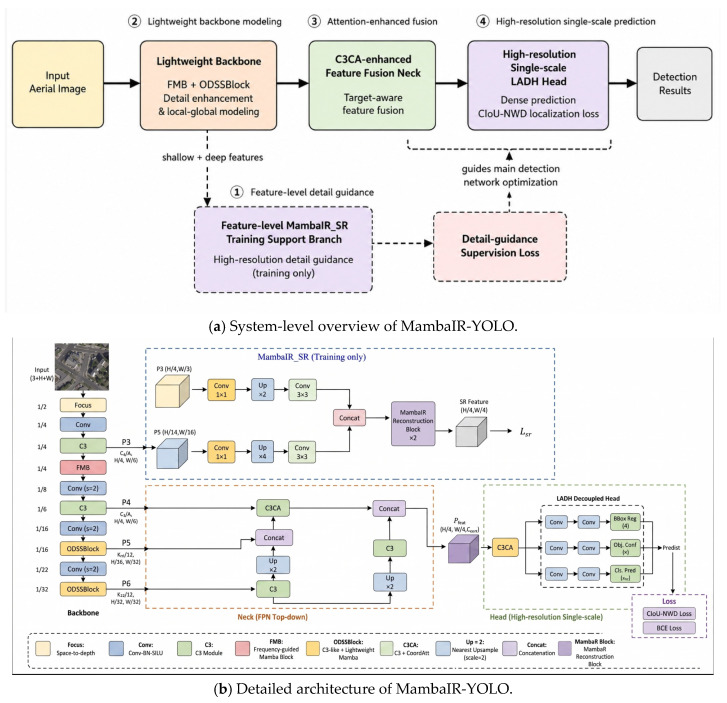
Overview and detailed architecture of MambaIR-YOLO.

To improve the comprehension of the proposed architecture, we first provide a system-level overview before presenting the detailed network structure. As shown in Figure 1a, MambaIR-YOLO consists of four main components: the feature-level MambaIR_SR training support branch, the FMB- and ODSSBlock-based lightweight backbone, the C3CA-enhanced feature fusion neck, and the high-resolution single-scale LADH detection head. Figure 1b further presents the detailed architecture of the proposed model.

Figure 1 illustrates the overall network architecture of the proposed MambaIR-YOLO. The model consists of a lightweight backbone network, a feature fusion neck, a high-resolution single-scale detection head, and a MambaIR_SR auxiliary branch used only during training. FMB and ODSSBlock are introduced into the backbone network to enhance shallow-layer detail representation and long-range dependency modeling, respectively. The Neck employs a top-down FPN feature propagation path [14] and performs single-scale dense predictions at the high-resolution feature layer. The MambaIR_SR branch is used only during the training phase to provide detail supervision and is removed during inference, enabling lightweight deployment.

In the backbone network section, this paper builds upon and improves the backbone output architecture of YOLOv5. The original YOLOv5 primarily relies on convolutional structures to extract features layer by layer, which struggles to preserve the fine-grained details of small objects [12]. This paper employs a lightweight Simple Stem for initial backbone output and introduces FMB into shallow backbone layers. FMB enhances shallow features using 3 × 3 depthwise convolution [34], Lightweight Mamba, and 1 × 1 projection.

In the mid-to-upper layers of the backbone, this paper introduces the ODSSBlock to perform joint local–global modeling of features. ODSSBlock embeds Lightweight Mamba into a C3-style block. The main branch performs state-space modeling, while the bypass branch preserves local details. This improves long-range modeling while preserving local details.

In the Neck section, this paper retains the top-down FPN semantic propagation path and fuses deep semantic features with shallow features via upsampling. Unlike the original three-scale detection output of YOLOv5 [12], this paper discards the low-resolution detection branch and performs single-scale dense prediction only at the high-resolution feature fusion layer. We empirically find that low-resolution detection heads contribute marginal improvements for extremely small objects in aerial scenes due to severe feature dilution.

Therefore, the overall framework of MambaIR-YOLO revolves around the main thread of “feature-level detail guidance—lightweight state-space modeling—high-resolution single-scale detection.” The MambaIR_SR branch addresses the issue of insufficient detail guidance for small targets during training; the ODSSBlock addresses the limited long-range context modeling capability of convolutional networks; the FMB addresses the issue of shallow-layer high-frequency details easily degrading before and after downsampling; and C3CA addresses the issue of insufficient response in key regions under complex backgrounds. Rather than being simply stacked, each module specifically addresses the distinct challenges in small-object detection in aerial remote sensing, collectively forming a lightweight hybrid model tailored for real-time detection.

During the training phase, the learning process of MambaIR-YOLO is formulated as:

(1) $$P_{\det}=H_{\det}(I_{rgb},I_{ir})$$

(2) $${\hat{I}}_{sr}=H_{SR}(F_{l1},F_{l2})$$

Here, $I_{rgb}$ and $I_{ir}$ denote the input images; $H_{\det}$ denotes the main detection network; and $P_{\det}$ denotes the detection features extracted by the detection backbone. $F_{l1}$ and $F_{l2}$ denote the shallow detail features and deep semantic features in the backbone network, respectively; $H_{SR}$ denotes the feature-level MambaIR_SR auxiliary mapping function; and ${\hat{I}}_{sr}$ denotes the high-resolution detail guidance results generated during the training phase.

During the inference phase, the MambaIR_SR auxiliary branch is removed, and the model retains only the main detection path to perform target prediction:

(3) $$Y=H_{\mathrm{head}}\left(H_{\mathrm{neck}}\left(H_{\mathrm{backbone}}(I)\right)\right)$$

Here, Backbone refers to the backbone network, Neck refers to the feature fusion network, Head refers to the high-resolution single-scale detection head, and Y represents the final detection results. As can be seen, the MambaIR_SR branch only provides detail guidance during the training phase and does not participate in the inference process of the final deployed model.

### 3.2. Feature-Level MambaIR_SR Training Support Branch

One of the main challenges in detecting small targets in aerial remote sensing lies in the low resolution of the targets themselves. In aerial image datasets such as VEDAI [37], vehicle targets typically occupy only a small area within the image, and information regarding their edges, corners, contours, and textures is extremely limited. After the input image undergoes successive convolutions and downsampling, the already weak high-frequency details of small targets are further attenuated [7]. This leads to unstable target responses in deep features, ultimately resulting in missed detections, false positives, and localization errors. Therefore, relying solely on feature fusion in the back end of the detection network cannot fully address the issue of insufficient detail in small targets. To provide the detection network with more comprehensive detail guidance before feature degradation occurs, this paper constructs a feature-level MambaIR_SR training auxiliary branch.

**Figure 2:**
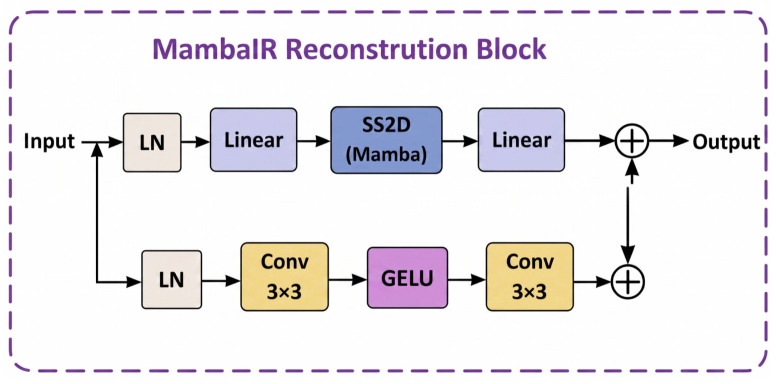
Structure diagram of the MambaIR_SR feature-level training auxiliary branch.

Figure 2 illustrates the architecture of the feature-level MambaIR_SR training auxiliary branch. Unlike traditional image-level super-resolution methods, this branch provides feature-level high-frequency supervision rather than performing image reconstruction [33,34]. Subsequently, a lightweight variant derived from the selective state-space model Mamba [22] is employed for feature modeling to obtain a discriminative detail-enhanced representation. This branch is used only during training for feature-level supervision and is removed during inference, incurring no additional computational overhead.

Let the shallow features in the backbone network be denoted as $F_{11}$, and the deep semantic features as $F_{12}$. Since the two have different spatial dimensions, we first upsample the deep features $F_{12}$ to the same spatial dimension as the shallow features $F_{11}$, resulting in the aligned deep features:

(4) $$F_{12}^{up}=Up(F_{12})$$

Here, Up refers to the upsampling operation, and $F_{12}^{up}$ refers to deep semantic features that match the dimensions of the shallow feature space after upsampling.

After aligning the spatial dimensions, the shallow-level detail feature $F_{11}$ is concatenated with the upsampled deep-level semantic feature $F_{12}^{up}$ along the channel dimension to obtain the multi-level fused feature $F_{cat}$:

(5) $$F_{cat}=\mathrm{Concat}(F_{11},F_{12}^{\mathrm{up}})$$

In this context, Concat refers to a channel-wise concatenation operation. This operation enables the auxiliary branch to simultaneously leverage both shallow spatial details and deep semantic context, providing a more comprehensive feature foundation for subsequent detail reconstruction.

Since the number of feature channels is high after concatenation, direct state-space modeling would increase the computational load. Therefore, this paper uses a 1 × 1 convolution to perform channel compression on $F_{cat}$, resulting in the compressed fused features $F_{c}$:

(6) $$F_{c}={\mathrm{Conv}}_{1\times 1}(F_{cat})$$

In this context, ${\mathrm{Conv}}_{1\times 1}$ refers to a 1 × 1 convolution operation, and $F_{c}$ refers to the fusion of features after channel compression.

Next, the $F_{c}$ input is fed into the auxiliary mapping function $H_{SR}$, which consists of Lightweight Mamba and a convolutional reconstruction layer, to generate the high-resolution detail-guided result ${\hat{I}}_{sr}$:

(7) $${\hat{I}}_{sr}=H_{SR}(F_{c})$$

Here, $H_{SR}$ refers to the feature-level MambaIR_SR auxiliary mapping function, and ${\hat{I}}_{sr}$ refers to the generated high-resolution detail-guided results.

The branch is driven by intermediate feature representations and generates auxiliary RGB/IR reconstruction outputs only for training-time supervision; it is not used as an image-level super-resolution inference branch. It fuses shallow and deep features to improve detail representation and is removed during inference without increasing parameters or computational cost.

Therefore, the role of MambaIR_SR can be understood as feature-level detail guidance during the training phase. By imposing additional high-resolution detail constraints, it enables the backbone network to focus more on the edges, textures, and structural information of small targets during training, thereby enhancing the model’s ability to learn discriminative features for small targets in aerial remote sensing. During the inference phase, the model does not need to execute this auxiliary branch, thus maintaining a lightweight, single-stage detection process.

### 3.3. ODSSBlock Local–Global State-Space Modeling Module

Traditional convolutional neural networks have strong local backbone output capabilities and can effectively capture object edges, textures, and local spatial structures. However, convolutional operations inherently rely on local receptive fields and have limited ability to model long-range dependencies [8,18]. For small-target detection in aerial remote sensing, where targets are small and the background is extensive, models must not only focus on the vehicle’s own limited local visual cues but also utilize contextual information from the surrounding scene—such as roads, buildings, and parking areas—to distinguish targets from complex background interference. If a network relies solely on standard convolutional structures, it struggles to adequately model the relationship between targets and distant backgrounds; conversely, directly incorporating global attention modules, such as those found in Transformers, may introduce significant computational overhead, hindering lightweight deployment [29,30].

To address the above issues, we propose the ODSSBlock local–global state-space modeling module. Based on the selective state-space modeling principle of Mamba [25,26], this module introduces lightweight state-space modeling capabilities into a C3-like dual-branch architecture, enabling the network to enhance long-range context representation while maintaining low computational complexity. ODSSBlock combines convolution and Mamba in a dual-branch design.

**Figure 3:**
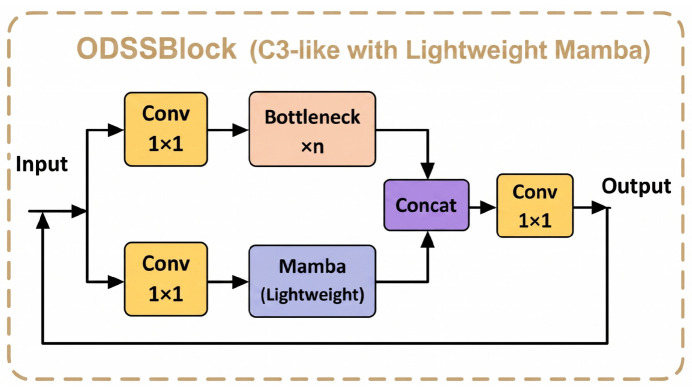
Schematic diagram of the ODSSBlock and lightweight Mamba architectures.

As shown in Figure 3, the ODSSBlock consists of a Lightweight Mamba main branch, a bypass retention branch, feature concatenation, and channel fusion. Given an input feature X, the input feature is first channel-adjusted through two 1 × 1 convolution branches. Specifically, the main branch feature $X_{1}$ is fed into a state-space modeling branch composed of n Lightweight Mamba units to enhance contextual representation, while the bypass branch feature $X_{2}$ is used to preserve the basic local information in the input. Subsequently, the outputs of the main and bypass branches are concatenated along the channel dimension, and channel fusion is performed via a 1 × 1 convolution to obtain the ODSSBlock’s output feature Y.

(8) $$X_{1}={\mathrm{Conv}}_{1\times 1}(X)$$

(9) $$X_{2}={\mathrm{Conv}}_{1\times 1}(X)$$

(10) $$X_{m}=M_{L}^{n}(X_{1})$$

(11) $$Y={\mathrm{Conv}}_{1\times 1}(\mathrm{Concat}(X_{m},X_{2}))$$

Here, X represents the input features of the ODSSBlock; $X_{1}$ represents the features entering the main branch of the Lightweight Mamba; $X_{2}$ represents the features entering the bypass branch; $M_{L}^{n}$ represents the state-space modeling branch consisting of n Lightweight Mamba units; $X_{m}$ represents the output features of the main branch; and Y represents the output features of the ODSSBlock.

Lightweight Mamba employs a channel-bottleneck design to reduce the computational overhead of state-space modeling. For input features, the number of channels is first reduced to a lower dimension using a 1 × 1 convolution, and then the two-dimensional feature map is flattened into a sequence format and fed into the Mamba module for state-space modeling [23,25]. After sequence modeling is complete, the sequence features are restored to a two-dimensional feature map, and the channel dimension is restored via a 1 × 1 convolution. Finally, a residual connection is used to add the output features to the input features, preserving local details and improving gradient stability.

(12) $$Z={\mathrm{Conv}}_{s}(X)$$

(13) $$Z_{m}=M_{\mathrm{Mamba}}(\mathrm{Flatten}(Z))$$

(14) $$Y_{m}={\mathrm{Conv}}_{e}(\mathrm{Reshape}(Z_{m}))+X$$

In this context, ${\mathrm{Conv}}_{s}$ refers to channel-compression convolution, Flatten refers to flattening a two-dimensional feature map into a sequence, Mamba refers to a state-space modeling operation, Reshape refers to restoring the sequence features to a two-dimensional feature map, ${\mathrm{Conv}}_{e}$ refers to channel-restoration convolution, and $Y_{m}$ refers to the output features of Lightweight Mamba.

ODSSBlock improves global modeling while preserving local details. In the overall network architecture of this paper, the ODSSBlock is embedded in the mid-to-deep stages of the backbone to enhance the contextual expressiveness of mid-to-high-level features. The MambaIR_SR branch primarily provides guidance for detail enhancement during the training phase, while the ODSSBlock further enhances feature representation capabilities within the backbone network. The two components complement each other: MambaIR_SR focuses on mitigating the lack of high-frequency details caused by the low resolution of small targets, whereas the ODSSBlock focuses on enhancing the ability to model local–global context in the feature space. Through this combination, the model is able to simultaneously capture richer local details and more stable semantic context during the backbone output process.

### 3.4. FMB Shallow Feature Modulation Module

Effective discriminative information for small targets in aerial remote sensing is typically concentrated in edges, textures, and spatial location responses within shallow-layer features. As the backbone network downsamples layer by layer, the spatial resolution of feature maps continuously decreases, and the inherently limited high-frequency details of small targets are easily compressed or even lost [6,7]. Once degraded, shallow features are difficult to recover in later stages. Therefore, modulating shallow features early is important for small-object detection.

To address this, this paper designs the FMB shallow-layer feature modulation module and deploys it in the shallow layers of the backbone network. Its design objective is not to fuse features from different levels but to pre-enhance high-frequency information—such as edges and textures—in shallow-layer features before these features enter the deep-layer downsampling process.

**Figure 4:**
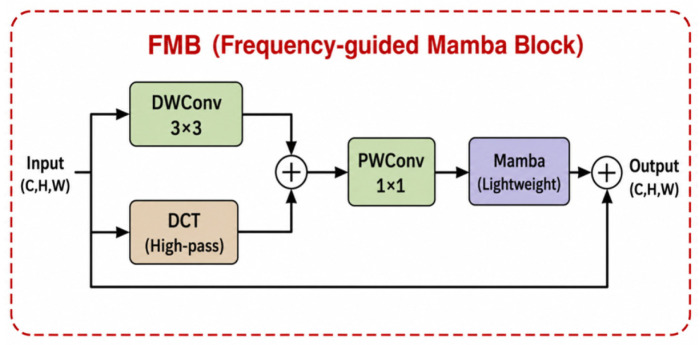
Block diagram of the FMB shallow feature modulation module.

As shown in Figure 4, the FMB adopts a frequency-guided residual structure consisting of a local spatial enhancement branch, a frequency-domain high-pass branch, a pointwise projection layer, and a Lightweight Mamba unit. Given the input feature $X_{f}$, the 3 × 3 depthwise convolution [38] branch is first used to extract local spatial details, such as edges, textures, and small structural responses. In parallel, a DCT-based high-pass branch is introduced to emphasize high-frequency components that are closely related to weak small-object details. The outputs of these two branches are then added to obtain the frequency-enhanced shallow feature representation:

(15) $$F_{d}={\mathrm{DWConv}}_{3\times 3}\left(X_{f}\right),\;F_{h}={\mathrm{DCT}}_{hp}\left(X_{f}\right),\;F_{s}=F_{d}+F_{h}$$

Here, $X_{f}$ denotes the input feature of FMB, ${\mathrm{DWConv}}_{3\times 3}(\cdot )$ denotes the 3 × 3 depthwise convolution, ${\mathrm{DCT}}_{hp}\left(\cdot \right)$ denotes the DCT-based high-pass filtering operation, $F_{d}$ and $F_{h}$ represent the local spatial feature and the high-frequency feature, respectively, and $F_{s}$ denotes their fused feature.

After the local spatial feature and the frequency-domain high-pass feature are fused, a 1 × 1 pointwise convolution is used for channel projection and feature integration. This operation adjusts the channel representation of the fused shallow feature and prepares it for subsequent lightweight state-space modeling:

(16) $$F_{p}={\mathrm{PWConv}}_{1\times 1}\left(F_{s}\right)$$

where ${\mathrm{PWConv}}_{1\times 1}\left(\cdot \right)$ denotes the 1 × 1 pointwise convolution, and $F_{p}$ denotes the projected feature after channel integration.

The projected feature is then fed into the Lightweight Mamba [22] unit to model long-range spatial dependencies with low computational overhead. Finally, the original input feature $X_{f}$ is forwarded through a unidirectional residual path to the final addition node, where it is added to the output of Lightweight Mamba to preserve shallow-layer information and stabilize feature propagation:

(17) $$Y_{f}=X_{f}+\mathrm{LMamba}\left(F_{p}\right)$$

where $\mathrm{LMamba}\left(\cdot \right)$ represents the Lightweight Mamba unit, and $Y_{f}$ denotes the output feature obtained by adding the transformed feature to the input feature $X_{f}$ at the final addition node.

Through this design, FMB first enhances local spatial details and high-frequency components in shallow features, then performs lightweight long-range dependency modeling through Lightweight Mamba, and finally preserves the original shallow representation through a unidirectional residual shortcut from the input to the final addition node. Therefore, the revised calculation process of Equations (15)–(17) is consistent with the structure illustrated in Figure 4, namely: parallel DWConv and DCT high-pass filtering, feature addition, pointwise projection, Lightweight Mamba modeling, and residual output.

The advantages of FMB are primarily reflected in two aspects. First, as a shallow frequency-guided modulation module at the front end of the backbone network, FMB enhances both local spatial details and high-frequency components before successive downsampling, thereby reducing the risk of severe information loss in subsequent deep layers. This is particularly important for small aerial objects, whose discriminative cues are mainly contained in weak edges, textures, and local structural responses. Second, FMB avoids the feature alignment overhead associated with complex cross-scale fusion. Instead, it achieves lightweight shallow-feature enhancement through depthwise convolution, DCT-based high-pass filtering, pointwise projection, Lightweight Mamba modeling, and residual feature preservation. As a result, the quality of shallow-layer features can be improved while maintaining low computational complexity.

In the overall network architecture of this paper, FMB, ODSSBlock, and C3CA have clearly defined and complementary roles. FMB is deployed in the shallow layers of the backbone and is responsible for enhancing local spatial details and high-frequency components before successive downsampling. ODSSBlock is inserted into the middle and upper layers of the backbone to strengthen local–global context modeling in deeper feature representations. C3CA is introduced at the Neck fusion stage to highlight target-relevant channel and spatial responses in complex backgrounds. By improving feature representation at different stages, these three components enable the network to preserve shallow-layer details, exploit mid-to-deep semantic context, and enhance key responses during feature fusion. Therefore, FMB focuses on early-stage detail preservation, ODSSBlock focuses on mid-to-deep semantic and contextual modeling, and C3CA focuses on fusion-stage attention enhancement, forming a clear division of labor between low-level detail enhancement, high-level context modeling, and target-aware feature fusion.

### 3.5. C3CA-LADH Fusion Enhancement and High-Resolution Single-Scale Detection Head

In aerial remote sensing images, small targets are often intermixed with complex background areas such as roads, buildings, shadows, and vegetation, resulting in weak target responses and strong background interference. Although deep neural networks can progressively extract semantic information, false positives and false negatives caused by background interference may still occur during the feature fusion stage if the regions associated with small targets cannot be effectively highlighted. To address this, this paper introduces the C3CA module into the top-down fusion node of the Neck architecture to enhance the response of key channels and spatial locations in the fused features. Additionally, the C3CA module is further integrated with the LADH [39] lightweight decoupling structure within the detection head, enabling the detection head to perform bounding box regression, target confidence prediction, and class prediction separately at the high-resolution feature layer.

**Figure 5:**
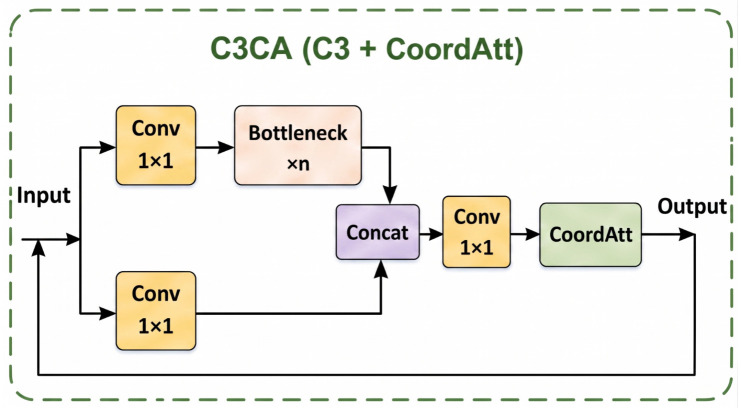
Schematic diagram of the C3CA-LADH high-resolution single-scale detection head.

As shown in Figure 5, C3CA introduces a coordinate attention [17] mechanism based on a C3-like architecture. Given the input fusion feature F, the input feature is first split into two branches: the main branch undergoes feature transformation via a 1 × 1 convolution and a bottleneck structure, while the bypass branch preserves the base information through a 1 × 1 convolution. Subsequently, the outputs of the two branches are concatenated along the channel dimension and undergo channel fusion via a 1 × 1 convolution. Finally, the coordinate attention mechanism is introduced to perform directional and positional encoding as well as channel recalibration on the fused features.

(18) $$F_{1}=B({\mathrm{Conv}}_{1\times 1}(F))$$

(19) $$F_{2}={\mathrm{Conv}}_{1\times 1}(F)$$

(20) $$F_{c3}={\mathrm{Conv}}_{1\times 1}(\mathrm{Concat}(F_{1},F_{2}))$$

(21) $$F_{ca}=\mathrm{CoordAtt}(F_{c3})$$

Here, F denotes the input-fused features; $F_{1}$ and $F_{2}$ denote the outputs of the main branch and the bypass branch in the C3-like structure, respectively; $F_{c3}$ denotes the features after channel fusion; CoordAtt denotes the coordinate attention operation; and $F_{ca}$ denotes the output features of C3CA.

Compared to conventional channel attention, coordinate attention preserves spatial position information in both horizontal and vertical directions while enhancing channel responses, making it more suitable for small-object localization tasks [21]. In aerial remote sensing images, vehicle targets are relatively small; if the attention mechanism only performs global channel re-calibration, it may overlook precise spatial locations. In contrast, coordinate attention, through direction-aware spatial encoding, enables the model to better focus on the regions where small targets are located, improving detection stability in complex backgrounds.

In terms of detector head design, this paper discards the original three-scale detection outputs of YOLOv5 [12] and retains only the single-scale detector head on the high-resolution fused feature layer. Traditional multi-scale detection architectures predict objects of different sizes through feature layers of varying resolutions, making them suitable for general object detection scenarios where object scales vary significantly. However, in aerial vehicle detection tasks such as VEDAI, targets are typically small in size, and target responses in deep, low-resolution detection layers are prone to compression, making it difficult to provide precise localization information. In contrast, the high-resolution fused feature layer contains richer spatial position information and, through the FPN [14], obtains deep semantic information, making it more suitable for the dense prediction of small targets.

To further enhance the detection head’s ability to distinguish small targets, this paper introduces the C3CA and LADH lightweight decoupling structures into the single-scale Detect model. First, the high-resolution fused features input to the detection head undergo channel adjustment via 1 × 1 convolutions, followed by C3CA to enhance target-relevant channels and spatial position responses. Based on this, the detection head is divided into a regression branch and a classification branch: the regression branch predicts bounding box positions, while the classification branch predicts target confidence and class probabilities. Both branches employ LADH blocks for lightweight spatial modeling. The LADH block consists of 3 × 1 and 1 × 3 asymmetric convolutions, which can expand the local receptive field and enhance structural awareness in both horizontal and vertical directions with low computational cost, making it suitable for modeling the shapes and boundaries of small objects, such as vehicles, in aerial images [39].

The prediction process of the detection head can be expressed as follows:

(22) $$F_{h}=C3\mathrm{CA}(\mathrm{Stem}(F_{in}))$$

(23) $$F_{reg}={\mathrm{LADH}}_{reg}(F_{h})$$

(24) $$F_{cls}={\mathrm{LADH}}_{cls}(F_{h})$$

(25) $$Y=\mathrm{Concat}(P_{reg}(F_{reg}),P_{obj}(F_{cls}),P_{cls}(F_{cls}))$$

In this context, $F_{in}$ refers to the high-resolution fused features from the input detection head; Stem refers to the 1 × 1 channel-adjusted convolution; $F_{h}$ refers to the detection features enhanced by C3CA; ${\mathrm{LADH}}_{reg}$ and ${\mathrm{LADH}}_{cls}$ refer to the LADH Blocks in the regression branch and classification branch, respectively; $P_{reg}$, $P_{obj}$, and $P_{cls}$ refer to the bounding box regression, object confidence, and class prediction layers, respectively; and Y represents the final prediction result.

In summary, the C3CA-LADH high-resolution single-scale detection head offers three key advantages. First, C3CA enhances the channel and spatial responses related to small objects, aiding in the localization of target regions in complex backgrounds. Second, the LADH decoupling structure optimizes features for regression and classification separately, thereby avoiding feature conflicts between different tasks. Third, single-scale high-resolution prediction mitigates the adverse effects of low-resolution detection layers on small-object localization while reducing the complexity of the detection branch. Together with the previously mentioned MambaIR_SR, ODSSBlock, and FMB, this detection head forms the lightweight, improved framework for aerial remote sensing described in this paper.

### 3.6. Loss Functions and Training Strategies

The training objective of MambaIR-YOLO consists of a detection loss and a super-resolution auxiliary loss during the training phase. The detection loss includes bounding box regression loss, object confidence loss, and classification loss. Unlike the original YOLOv5, which uses only IoU-based losses for bounding box regression, this paper introduces the Normalized Gaussian Wasserstein Distance (NWD) [40] into the localization loss and performs a weighted fusion with CIoU [41] to enhance the model’s robustness to bounding box perturbations for small aerial targets.

Vehicle targets in aerial remote sensing images are typically small in size. When the width and height of the target bounding box are small, even slight offsets between the predicted box and the ground-truth box can lead to a significant drop in IoU [39,42,43], making traditional IoU loss relatively sensitive to localization errors for small targets. NWD models the bounding box as a two-dimensional Gaussian distribution and measures the distribution difference between the predicted and ground-truth boxes using the normalized Wasserstein distance, thereby mitigating the severe impact of minor shifts in small-target boxes on the localization loss. Therefore, this paper adopts a CIoU-NWD hybrid localization loss to improve the stability of small-target bounding box regression.

Let the predicted box be $B_{p}=\left(x_{p},y_{p},w_{p},h_{p}\right)$ and the true box be $B_{t}=\left(x_{t},y_{t},w_{t},h_{t}\right)$; the Wasserstein distance between the two can be expressed as:

(26) $$W_{2}^{2}(B_{p},B_{t})={\left(x_{p}-x_{t}\right)}^{2}+{\left(y_{p}-y_{t}\right)}^{2}+\frac{{\left(w_{p}-w_{t}\right)}^{2}+{\left(h_{p}-h_{t}\right)}^{2}}{4}$$

NWD can be expressed as:

(27) $$\mathrm{NWD}(B_{p},B_{t})=\exp\left(-\frac{\sqrt{W_{2}^{2}(B_{p},B_{t})}}{C}\right)$$

Here, C is a normalization constant used to control the scale of distance normalization. The NWD ranges from 0 to 1; a higher value indicates that the predicted bounding box is closer to the ground-truth bounding box.

This paper performs a weighted fusion of CIoU and NWD to obtain the bounding box regression loss:

(28) $$L_{\mathrm{box}}=\mathrm{mean}[\alpha (1-\mathrm{CIoU})+(1-\alpha )(1-\mathrm{NWD})]$$

Here, α represents the fusion weight between the CIoU loss and the NWD-based localization loss. In this study, α is set to 0.5 to assign equal importance to the two complementary localization constraints. Specifically, CIoU focuses on the geometric consistency between the predicted bounding box and the ground-truth bounding box, including overlap area, center distance, and aspect-ratio consistency, whereas NWD is less sensitive to slight coordinate deviations and provides more stable localization supervision for extremely small objects. If α is too large, the localization loss is dominated by CIoU, which may still be overly sensitive to small spatial offsets in tiny bounding boxes. Conversely, if α is too small, the geometric overlap constraint provided by CIoU may be weakened. Therefore, α = 0.5 is adopted as a balanced setting to jointly preserve the general bounding-box regression capability of CIoU and the small-object localization robustness of NWD. This setting follows the principle of equal weighting when no prior preference is assigned to either loss component.

The object confidence loss and classification loss use the BCEWithLogitsLoss from the YOLO series of detectors. The detection loss can be expressed as:

(29) $$L_{\det}=\lambda_{\mathrm{box}}L_{\mathrm{box}}+\lambda_{\mathrm{obj}}L_{\mathrm{obj}}+\lambda_{\mathrm{cls}}L_{\mathrm{cls}}$$

Here, $L_{\mathrm{box}}$ denotes the CIoU-NWD hybrid bounding box regression loss, $L_{\mathrm{obj}}$ denotes the object confidence loss, and $L_{\mathrm{cls}}$ denotes the classification loss; $\lambda_{\mathrm{box}}$, $\lambda_{\mathrm{obj}}$, and $\lambda_{\mathrm{cls}}$ represent the weight coefficients for these three types of losses, respectively.

To enhance the model’s ability to perceive detailed information on small aerial targets, this paper introduces MambaIR_SR auxiliary supervision during the training phase. This auxiliary branch utilizes shallow detail features and deep semantic features to generate high-resolution detail-guided results and constrains its output via L1 loss, enabling the main network to focus more on the edges, textures, and structural information of small targets during training. Since this paper uses both RGB and infrared images as inputs, in the RGB + IR + MF mode, the MambaIR_SR output consists of an RGB reconstruction component and an infrared reconstruction component. The RGB component is aligned with the original visible-light image, while the infrared component is aligned with the original infrared image.

The super-resolution auxiliary loss during the training phase can be expressed as:

(30) $$L_{\mathrm{sr}}=\beta \left(\|{\hat{I}}_{\mathrm{sr}}^{\mathrm{rgb}}-I_{\mathrm{rgb}}{\|}_{1}+\|{\hat{I}}_{\mathrm{sr}}^{\mathrm{ir}}-I_{\mathrm{ir}}{\|}_{1}\right)$$

Here, ${\hat{I}}_{\mathrm{sr}}^{\mathrm{rgb}}$ represents the RGB reconstruction component of the MambaIR_SR output, ${\hat{I}}_{\mathrm{sr}}^{\mathrm{ir}}$ represents the infrared reconstruction component of the output, $I_{\mathrm{rgb}}$ and $I_{\mathrm{ir}}$ denote the original RGB image and infrared image, respectively, and β represents the auxiliary loss weight.

Here, β denotes the weight coefficient of the MambaIR_SR auxiliary loss. In this study, β is set to 0.1 because the auxiliary branch is intended to provide supplementary high-frequency detail guidance rather than replace the primary detection objective. A relatively small weight keeps the detection loss dominant during training, while still allowing the MambaIR_SR branch to encourage the backbone to learn edge, texture, and structural information of small objects. If β is too large, the model may be overly optimized toward RGB and infrared reconstruction, potentially weakening object classification and localization. If β is too small, the auxiliary supervision may become ineffective. Therefore, β = 0.1 is adopted as a conservative and balanced setting for auxiliary detail supervision.

Therefore, the total training loss of the model can be expressed as:

(31) $$L_{\mathrm{total}}=L_{\det}+L_{\mathrm{sr}}$$

Here, $L_{\mathrm{total}}$ represents the total model loss. The detection loss is responsible for optimizing target classification, target confidence, and bounding box regression; the super-resolution auxiliary loss provides detail guidance during the training phase, enabling the network to focus more on the texture, edges, and structural information of small aerial targets.

It is important to note that the MambaIR_SR branch is involved in optimization only during the training phase. Once training is complete, this branch does not participate in the inference process. The model used during inference consists solely of the backbone network, the Neck, and the high-resolution single-scale detection head. Therefore, the detail enhancement provided by the auxiliary branch is primarily reflected in the learning process during training and does not increase the number of parameters, GFLOPs, or inference time of the final deployed model.

## 4. Experiments and Analysis of Results

### 4.1. Datasets and Evaluation Metrics

To validate the effectiveness of the MambaIR-YOLO method proposed in this paper for small-object detection in aerial remote sensing, we conducted experiments using the VEDAI [38] aerial image dataset. The VEDAI dataset is primarily designed for vehicle object detection in aerial remote sensing scenarios. The images were captured by high-altitude platforms and are characterized by a typical bird’s-eye view, a high density of small objects, complex backgrounds, and significant variations in object scale. Compared to typical object detection datasets in natural scenes, the vehicle targets in the VEDAI dataset generally occupy a small portion of the image, have limited visual details, and are easily obscured by background elements such as roads, buildings, shadows, vegetation, and other terrain features. As such, this dataset is well-suited for evaluating the model’s performance in small-object detection tasks in aerial remote sensing.

**Figure 6:**
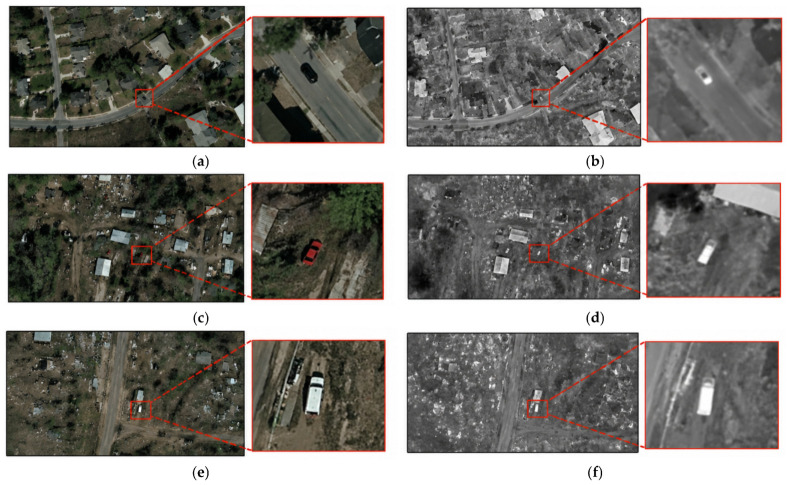
Examples of RGB and infrared images from the VEDAI dataset. (**a**,**c**,**e**) are RGB images; (**b**,**d**,**f**) are the corresponding infrared images.

As shown in Figure 6, vehicle targets in the VEDAI dataset generally exhibit characteristics such as small size, weak texture, and indistinct boundaries. In complex backgrounds, some vehicle targets bear a high resemblance to road textures, building edges, or shadowed areas, which can easily lead to false positives in detection models. At the same time, because aerial images have a wide field of view, a single image may contain multiple small-scale and sparsely distributed vehicle targets simultaneously. The model must accurately locate the targets within complex backgrounds and distinguish between target classes. These characteristics place high demands on the detection model’s ability to extract features from small targets, suppress background noise, and perform lightweight inference.

In this study, the input image size was uniformly adjusted to 512 × 512 for the experiments, and model training and performance evaluation were conducted by dividing the data into training and testing sets; all comparison methods were evaluated using the same dataset, the same input size, and the same evaluation metrics.

To further validate the robustness and generalization capabilities of MambaIR-YOLO across different aerial remote sensing scenarios, data modalities, and extremely small targets, this paper introduces three mainstream public remote sensing datasets to conduct cross-scenario generalization experiments:

1. DroneVehicle Dataset [44]: This is a large-scale, dual-modal (infrared and visible light) vehicle detection dataset captured from a drone’s perspective. It contains a vast number of dense vehicle scenes collected during both daytime and nighttime and is used to evaluate the model’s multimodal fusion and generalization performance on a larger scale of dual-modal data.
2. AI-TOD Dataset [45]: This is a monomodal visible-light dataset specifically designed for aerial imagery containing a massive number of extremely small targets, with an average target size of only 12.8 pixels. This dataset is used to pose an extreme challenge and to validate the ability of the MambaIR_SR super-resolution auxiliary branch proposed in this paper to mitigate the problem of early-stage detail degradation.
3. DOTA Dataset [1]: As a large-scale satellite remote sensing image dataset, DOTA covers an extremely wide range of scales. Since this paper adopts a high-resolution single-scale prediction strategy, this generalization experiment primarily focuses on typical small-object categories in DOTA (such as cars, trucks, and ships) to evaluate the spatial localization limits of the single-scale dense prediction head against complex ground object backgrounds.

The experimental implementation was conducted under the following settings. All experiments were conducted on an Ubuntu 22.04.3 LTS operating system. The model was implemented using Python 3.10.20 and PyTorch 2.1.0 with CUDA 12.1 and cuDNN 8.9.2 acceleration. The experiments were performed on a workstation equipped with an NVIDIA GeForce RTX 4090D GPU (Santa Clara, CA, USA). In terms of evaluation metrics, this paper employs metrics commonly used in object detection tasks—such as Precision, Recall, mAP50, the number of parameters, computational cost, and inference speed—to conduct a comprehensive evaluation of the model [4,5]. Among these, Precision represents the proportion of true objects among the bounding boxes predicted by the model as positive samples, primarily reflecting the model’s false positives; Recall represents the proportion of true objects correctly detected by the model, primarily reflecting the model’s false negatives. These two metrics can be expressed as follows:

(32) $$\mathrm{Precision}=\frac{TP}{TP+FP}$$

(33) $$\mathrm{Recall}=\frac{TP}{TP+FN}$$

In this context, TP refers to the number of correctly detected targets, FP refers to the number of background objects incorrectly detected as targets, and FN refers to the number of true targets that were not detected. A higher Precision indicates fewer false positives, while a higher Recall indicates fewer false negatives. In the detection of small targets in aerial remote sensing, due to the complex background and small target sizes, models are prone to false positives and false negatives; therefore, Precision and Recall provide complementary perspectives on the model’s detection performance.

Average Precision (AP) is a key metric for evaluating single-class object detection performance and is calculated from the area under the Precision–Recall curve. For multi-class detection tasks, mean Average Precision (mAP) is obtained by averaging the AP values across all object categories. In this study, both mAP50 and mAP50–95 are employed to evaluate detection accuracy. Specifically, mAP50 denotes the mean AP at an Intersection over Union (IoU) threshold of 0.5 and reflects the overall object recognition and localization capability of the detector under a relatively permissive matching criterion. In contrast, mAP50–95 denotes the mean AP averaged over IoU thresholds ranging from 0.50 to 0.95 with a step size of 0.05. Because it evaluates detection performance across progressively stricter localization criteria, mAP50–95 provides a more comprehensive assessment of bounding-box localization quality and detection robustness.

(34) $$\mathrm{mAP}=\frac{1}{N}\sum_{i=1}^{N}AP_{i}$$

Here, N represents the number of detection classes, and $AP_{i}$ represents the average precision for the *i*-th class.

In addition to detection accuracy, this paper also evaluates model complexity and inference efficiency. The number of parameters (Params) measures the number of trainable parameters in a model and is typically expressed in M. The smaller the number of parameters, the lower the model’s storage requirements, making it more suitable for deployment on resource-constrained devices. Computational load (GFLOPs) measures the number of floating-point operations required for the model to complete a single forward pass and is typically expressed in G. The lower the GFLOPs value, the lower the model’s theoretical computational overhead. Inference speed (FPS) indicates the number of images the model can process per second and directly reflects the model’s real-time performance in practical applications. Overall, this paper employs Precision, Recall, mAP50, mAP50–95, Params, GFLOPs, and FPS to comprehensively evaluate the detection accuracy, localization robustness, model complexity, and inference efficiency of MambaIR-YOLO.

### 4.2. Experimental Setup

To ensure the fairness of the experimental results, all models in this paper were trained and tested under the same experimental conditions. The experiments were implemented using the PyTorch 2.1.0 deep learning framework with CUDA 12.1 acceleration, with MambaIR-YOLO built on the YOLOv5 detection framework., with MambaIR-YOLO built on the YOLOv5 detection framework. All models were evaluated using the same data partitioning method, input dimensions, and evaluation metrics to ensure consistency in the comparison of different methods.

In the experiments described in this paper, the input image size was uniformly set to 512 × 512, with an input mode of RGB + IR + MF. The training batch size was set to 2, the number of training epochs to 300, and the SGD optimizer was used with an initial learning rate of 0.01, momentum of 0.937, and weight decay of 0.0005. Warmup epochs were set to 3.0, with box, obj, and cls loss weights of 0.05, 1.0, and 0.5, respectively, and the SR auxiliary loss weight set to 0.1; during the training phase, the MambaIR_SR auxiliary branch was enabled to constrain the RGB and infrared reconstruction results via L1 loss, while during the inference phase, this branch was removed, retaining only the main detection path to complete object prediction.

The experiments in this paper primarily evaluate the model in terms of detection accuracy, model complexity, and inference efficiency. For detection accuracy, Precision, Recall, mAP50, and mAP50–95 are used as the primary metrics. Precision reflects the model’s ability to suppress false-positive predictions, whereas Recall measures its ability to detect true objects. mAP50 evaluates average detection performance at an IoU threshold of 0.5, while mAP50–95 further assesses localization robustness across multiple IoU thresholds from 0.50 to 0.95.

Regarding model complexity, this paper uses the number of parameters (Params) and computational load (GFLOPs) as metrics. Params represents the number of trainable parameters in the model and reflects its memory overhead; GFLOPs represents the amount of floating-point computation required for the model to complete a single forward inference, reflecting the model’s theoretical computational complexity. It should be noted that the complexity statistics in this paper are based on the model structure in inference mode; the MambaIR_SR auxiliary branch is removed during the inference stage and is therefore not included in the final deployed model’s Params or GFLOPs.

In terms of inference efficiency, this paper uses the average inference latency per image and FPS as evaluation metrics. Average inference latency represents the time required for the model to process a single input image, while FPS represents the number of images the model can process per second. For real-time aerial remote sensing detection tasks, a high FPS ensures the model’s applicability in scenarios such as drone inspections, traffic monitoring, and real-time object recognition.

To reduce the influence of random initialization and stochastic optimization, the complete MambaIR-YOLO model was independently trained three times under identical experimental settings. Unless otherwise specified, the final performance of the complete model is reported as the mean ± standard deviation over the three independent runs.

### 4.3. Comparison with Mainstream Methods

To validate the effectiveness and competitiveness of the proposed MambaIR-YOLO for aerial small-object detection, we compare it with several representative and recent detection methods, including YOLOv8, YOLOv11, YOLO26, and SuperYOLO [19]. All models were trained and tested on the VEDAI dataset under the same input resolution of 512 × 512 and consistent experimental settings. To provide a comprehensive comparison of detection accuracy, model complexity, and inference efficiency, we report per-class AP, mAP50, mAP50–95, parameter count, GFLOPs, and FPS.

Table 1 compares MambaIR-YOLO with representative YOLO-based detectors and lightweight aerial object detection methods on the VEDAI dataset. The proposed method achieves the highest overall accuracy, with an mAP50 of 84.19% and an mAP50–95 of 51.47%, while requiring only approximately 4.45 M parameters and 19.97 GFLOPs. These results indicate that the performance gain is not obtained by simply increasing model scale; instead, the proposed framework maintains a compact parameter budget while improving both detection accuracy and localization robustness.

Compared with recent YOLO-based baselines, MambaIR-YOLO demonstrates particularly clear advantages in the aerial small-object setting. Relative to YOLOv8s, YOLO11s, and YOLO26s, the proposed method improves mAP50 by 18.09, 14.79, and 16.69 percentage points, respectively. The corresponding gains in mAP50–95 are 12.67, 11.27, and 11.77 percentage points. These improvements under the stricter mAP50–95 criterion are especially important because they indicate that the proposed method improves not only object recognition but also bounding-box localization robustness across multiple IoU thresholds. This advantage is consistent with the design objective of preserving high-resolution spatial information and strengthening weak small-object representations.

Compared with SuperYOLO [19], MambaIR-YOLO improves mAP50 from 75.09% to 84.19% and mAP50–95 from 43.72% to 51.47%, corresponding to gains of 9.10 and 7.75 percentage points, respectively, while reducing the parameter count from 4.84 M to approximately 4.45 M. Although the proposed method has a slightly higher computational cost and a lower FPS than SuperYOLO, it still achieves 196.36 FPS under the reported evaluation setting, indicating that real-time inference capability is retained. Therefore, the advantage of MambaIR-YOLO lies not in maximizing a single efficiency metric but in achieving a favorable balance among small-object detection accuracy, localization robustness, model compactness, and real-time inference speed.

### 4.4. Ablation Experiment

To further evaluate the effectiveness of the proposed components, we conducted controlled ablation experiments on the VEDAI dataset. Starting from the complete MambaIR-YOLO framework, the feature-level MambaIR_SR training auxiliary branch, ODSSBlock local–global state-space modeling module, FMB shallow feature modulation module, and C3CA fusion enhancement module were removed individually while the remaining training and evaluation settings were kept unchanged. In addition, the original YOLOv5s model was trained and evaluated under the same experimental settings to provide a direct baseline for assessing the overall effectiveness of the proposed architectural improvements. Both mAP50 and mAP50–95 were reported to assess the contribution of each component from complementary perspectives: mAP50 reflects overall detection capability at an IoU threshold of 0.5, whereas mAP50–95 provides a stricter evaluation of localization robustness across multiple IoU thresholds. This controlled comparison enables a more comprehensive analysis of how each component contributes to small-object representation, contextual modeling, and precise localization in aerial remote sensing imagery.

Table 2 compares the original YOLOv5s baseline, the proposed MambaIR-YOLO, and its four ablation variants. Under the same dataset split, input resolution, training schedule, and evaluation protocol, the original YOLOv5s achieves an mAP50 of 56.69% and an mAP50–95 of 31.70%, whereas the complete MambaIR-YOLO achieves 84.19% and 51.47%, respectively. This corresponds to improvements of 27.50 percentage points in mAP50 and 19.77 percentage points in mAP50–95, demonstrating the substantial overall advantage of the proposed framework over the original baseline. Removing any individual component results in performance degradation under both evaluation criteria, indicating that the proposed modules provide complementary contributions rather than isolated improvements. Notably, the consistent decreases in mAP50–95 further show that these components contribute not only to object recognition at IoU = 0.5 but also to more robust bounding-box localization under stricter overlap thresholds.

First, removing the MambaIR_SR training auxiliary branch decreases mAP50 from 84.19% to 80.00% and mAP50–95 from 51.47% to 48.80%, corresponding to reductions of 4.19 and 2.67 percentage points, respectively. This result indicates that training-time feature-level detail guidance improves not only object recognition under the IoU = 0.5 criterion but also localization robustness across stricter IoU thresholds. Because small aerial objects contain limited edge, texture, and structural cues, these weak features can be progressively attenuated during backbone downsampling. By integrating shallow detail features with deeper semantic information during training, the MambaIR_SR branch provides additional detail-oriented optimization signals and thereby improves the discriminability and localization stability of small objects.

Second, removing ODSSBlock reduces mAP50 from 84.19% to 80.28% and mAP50–95 from 51.47% to 49.84%, corresponding to decreases of 3.91 and 1.63 percentage points, respectively. This degradation confirms the importance of joint local–global context modeling for aerial small-object detection. ODSSBlock preserves local structural information through its dual-branch architecture while employing Lightweight Mamba to capture longer-range contextual dependencies. Such contextual information is particularly useful in complex aerial scenes, where small targets must be distinguished from visually similar background patterns such as roads, shadows, and building structures.

Third, removing FMB results in an mAP50 of 77.73% and an mAP50–95 of 48.73%, representing decreases of 6.46 and 2.74 percentage points compared with the complete model. The pronounced reduction in mAP50 suggests that shallow feature modulation plays an important role in preserving the weak visual responses of small targets before repeated downsampling. Meanwhile, the decline in mAP50–95 indicates that early enhancement of local edge and structural information also contributes to more stable localization under stricter IoU criteria.

Finally, removing C3CA causes the largest overall degradation, reducing mAP50 from 84.19% to 76.15% and mAP50–95 from 51.47% to 45.27%. These decreases of 8.04 and 6.20 percentage points indicate that attention-enhanced feature fusion is particularly important for the proposed high-resolution single-scale detection strategy. By strengthening target-relevant channel responses while retaining positional information, C3CA improves the discriminability of fused features in complex backgrounds and contributes substantially to both detection accuracy and precise localization.

Overall, the ablation results reveal complementary functional roles among the proposed components. MambaIR_SR primarily strengthens training-time detail guidance, ODSSBlock improves local–global contextual representation, FMB mitigates shallow-detail degradation, and C3CA enhances target-relevant responses during feature fusion. The consistent improvements in both mAP50 and mAP50–95 demonstrate that the complete framework improves not only object recognition but also localization robustness across different IoU criteria.

### 4.5. Analysis of Model Complexity

In addition to detection accuracy, model complexity is also a key metric for evaluating small-target detection methods in aerial remote sensing. UAV platforms, edge computing devices, and real-time monitoring systems are typically constrained by computational resources, graphics memory capacity, and power consumption. Therefore, detection models must not only achieve high detection accuracy but also maintain a low number of parameters and computational complexity. If a model is too complex, it will struggle to meet practical deployment requirements even if it achieves high detection accuracy. To this end, this paper conducts a comparative analysis of the parameter count, GFLOPs, and inference speed of different model variants to evaluate the computational characteristics and deployment efficiency of MambaIR-YOLO.

Table 3 compares the model complexity and inference efficiency of the original YOLOv5s baseline, the complete MambaIR-YOLO, and its ablation variants. Compared with YOLOv5s, the proposed model reduces the parameter count from 7.041 M to 4.458 M, corresponding to a reduction of approximately 36.69%. Although the proposed model requires higher theoretical computation, increasing from 15.80 to 19.97 GFLOPs, it still achieves 196.36 FPS under a batch size of 1 and therefore maintains real-time inference capability. These results indicate that MambaIR-YOLO substantially improves detection accuracy while reducing model size and preserving competitive inference efficiency. Meanwhile, the ablated variants exhibit different runtime characteristics, indicating that practical inference speed is influenced not only by parameter count and theoretical GFLOPs but also by operator composition, feature transformation patterns, and hardware execution efficiency. Overall, the results reveal a clear trade-off between feature modeling capability, computational complexity, and inference throughput.

It is worth noting that removing the MambaIR_SR branch does not change the parameter count or GFLOPs of the deployed model. This is consistent with the design of MambaIR_SR as a training-only auxiliary branch, which provides detail-oriented optimization during training but does not participate in the final detection path during inference. Therefore, the branch introduces no additional structural complexity into the deployed network. The small difference in measured FPS between the complete model and the w/o MambaIR variant reflects empirical runtime variation rather than a change in the theoretical inference graph. This design allows the model to benefit from training-time feature-level detail guidance without increasing the parameter count or computational cost of final deployment.

The remaining structural ablation variants exhibit different complexity and runtime characteristics. In particular, the w/o ODSSBlock variant achieves the highest FPS, indicating that the state-space modeling operations in ODSSBlock introduce additional runtime overhead despite their contribution to long-range contextual representation. Similarly, the w/o FMB and w/o C3CA variants achieve slightly higher inference speeds than the complete model, suggesting that shallow feature modulation and attention-enhanced fusion also incur moderate computational cost. However, as demonstrated by the accuracy ablation results in Table 2, removing these modules leads to noticeable degradation in mAP50 and mAP50–95. Therefore, ODSSBlock, FMB, and C3CA should be interpreted as accuracy-oriented enhancement components that trade a limited amount of inference throughput for improved contextual modeling, detail preservation, and feature discrimination.

Overall, MambaIR-YOLO achieves a favorable balance among detection accuracy, model complexity, and inference efficiency. Although some enhancement modules introduce additional runtime overhead, the complete model still maintains real-time throughput while delivering the best detection performance among the ablation variants. This accuracy–efficiency trade-off is particularly valuable for UAV-based and edge-oriented aerial remote sensing applications, where both recognition reliability and practical inference speed are essential.

### 4.6. Analysis of Inference Efficiency

In practical aerial remote sensing applications, detection models must not only achieve high accuracy but also meet real-time processing requirements. Scenarios such as UAV inspections, traffic monitoring, and emergency response typically require models to rapidly process continuous image or video streams; therefore, inference speed is a key metric for evaluating a model’s practicality. To further validate the deployment value of MambaIR-YOLO, this paper evaluates the model’s inference efficiency.

As shown in Table 4, under conditions of an input size of 512 × 512 and a batch size of 1, MambaIR-YOLO achieves an average inference latency of 5.093 ms/image, a P95 latency of 5.366 ms, an inference speed of 196.36 FPS, and a peak GPU memory usage of 371.81 MB. These results demonstrate that the method proposed in this paper maintains high detection accuracy while still offering excellent real-time inference capabilities.

The real-time performance of MambaIR-YOLO can be attributed to several architectural choices. First, the MambaIR_SR branch is used only during training and is completely removed from the deployed inference path, thereby avoiding additional inference-stage computation. Second, although ODSSBlock introduces state-space modeling overhead, its channel-compressed Lightweight Mamba design constrains the computational burden by performing long-range modeling in a reduced-dimensional feature space. Third, FMB and C3CA are implemented with relatively compact structures, limiting the additional cost of shallow feature enhancement and attention-based fusion. Finally, the high-resolution single-scale detection strategy avoids multiple prediction branches and helps maintain an efficient overall detection pipeline.

Combining Table 1, Table 3 and Table 4, MambaIR-YOLO demonstrates a favorable balance between detection accuracy, model complexity, and inference speed. The proposed model does not pursue maximum throughput alone; instead, it retains real-time processing capability while incorporating feature-level guidance, long-range context modeling, shallow detail preservation, and attention-enhanced fusion. This balance makes the framework suitable for real-time aerial small-object detection scenarios in UAV and edge-oriented applications.

### 4.7. Analysis of Visualization Results

To more intuitively verify the effectiveness of MambaIR-YOLO in detecting small targets in aerial remote sensing, this paper conducts a visual analysis of the model’s detection results. The visualization results include comparisons of detection results across different models, confusion matrices, and Precision–Confidence, Recall–Confidence, F1–Confidence, and Precision–Recall curves. The comparison of detection results is used to demonstrate the model’s ability to localize targets in complex scenes; the confusion matrix is used to analyze classification performance across different categories; and the evaluation curves are used to further observe the model’s detection stability at different confidence thresholds.

**Figure 7:**
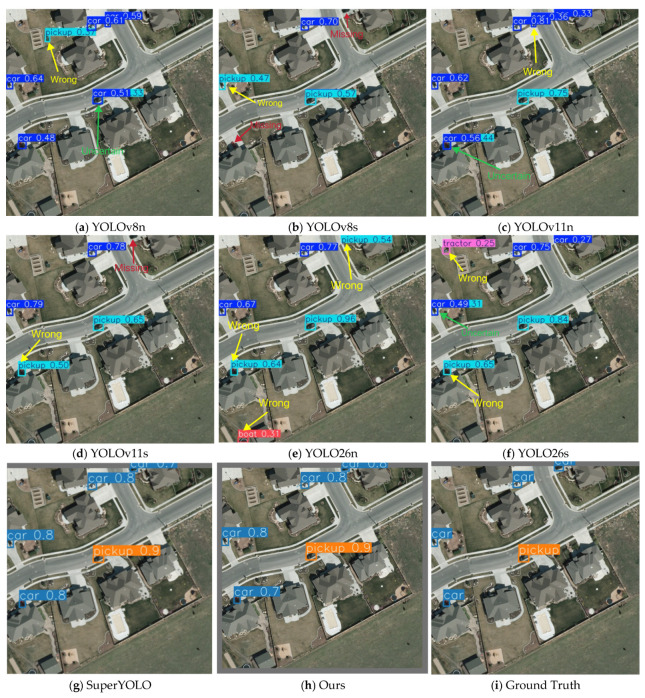
Comparison of detection results from different models on the VEDAI dataset.

Figure 7 shows a comparison of detection results from different models on the VEDAI dataset (additional qualitative comparisons are provided in Appendix A). The visualized samples were primarily selected from scenes featuring small-sized targets, complex backgrounds, dense targets, or low contrast to evaluate the model’s detection performance on challenging samples. To clearly show the advantages of the proposed method, Figure 7 provides a high-resolution visual comparison of MambaIR-YOLO and the latest state-of-the-art detectors (like YOLOv8, YOLOv11 and YOLO26) in challenging scenarios. As shown, even the most recent models, such as YOLOv11 and YOLO26, tend to miss detections (marked as ‘missing’) and make mistakes (marked as ‘wrong’) when handling complex backgrounds. Especially for very small vehicle targets, blurry boundaries, or those with visual features similar to road textures, these baseline models struggle to achieve accurate localisation and classification. In contrast, MambaIR-YOLO shows higher robustness, reliably detecting tiny vehicles with greater confidence while effectively suppressing background false positives. Consistent with the quantitative results in Table 1, this visual evidence confirms that the proposed method outperforms the latest YOLO variants in small-target localisation and background suppression in complex aerial remote sensing scenes.

In densely populated scenes, MambaIR-YOLO demonstrates superior ability to distinguish between adjacent small targets, thereby reducing missed detections caused by close target spacing. In low-contrast or shadow-interfered scenes, the method proposed in this paper is also capable of focusing more accurately on vehicle regions. This is primarily attributable to the enhanced learning of small-target details provided by the MambaIR_SR training auxiliary branch, the pre-enhancement of shallow-layer high-frequency textures and edge responses by FMB, the ability of ODSSBlock to model local–global contextual relationships, and the response enhancement for target-related regions by C3CA during the fusion stage.

**Figure 8:**
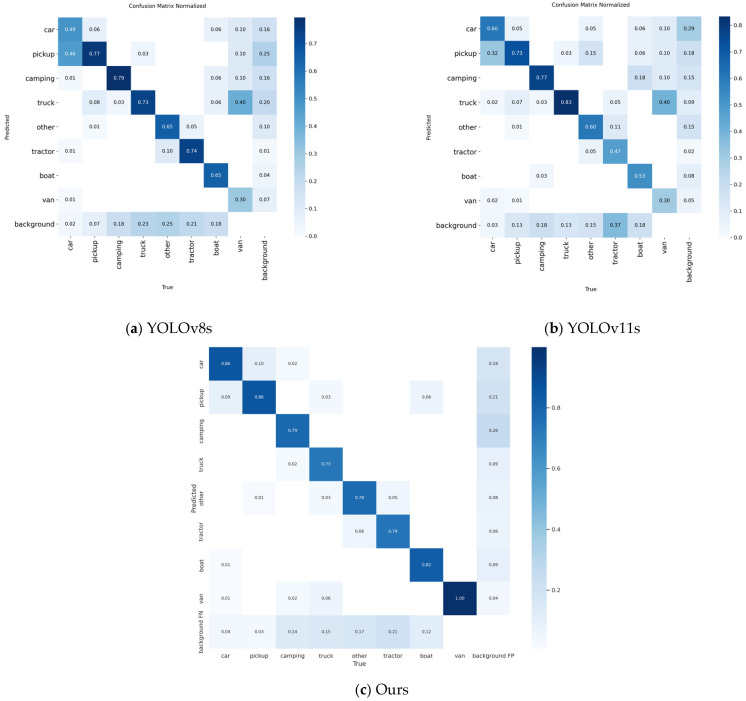
Comparison of confusion matrices between different models on the VEDAI dataset.

To further demonstrate the advantages of the proposed method in distinguishing difficult categories and suppressing background interference, we compared the confusion matrices of MambaIR-YOLO with other representative models (e.g., YOLOv8s and YOLOv11), as shown in Figure 8. As can be seen from Figure 8, MambaIR-YOLO exhibits significant advantages in accurate classification and background suppression. For instance, in identifying ‘car’ and ‘pickup’, our model achieves high accuracy of 0.86 and 0.86, respectively, outperforming YOLOv8s (0.60 and 0.73) and YOLOv11s (0.55 and 0.75). More importantly, for categories that are extremely easily confused with complex backgrounds, such as ‘van’ and ‘boat’, MambaIR-YOLO reaches remarkable accuracies of 1.00 and 0.82, which are vastly superior to the baseline models. These results suggest that the proposed framework provides stronger fine-grained discrimination and background suppression capabilities. Together with the ablation results in Table 2, the observations further support the effectiveness of the MambaIR_SR detail-guidance mechanism and ODSSBlock-based contextual modeling. Although challenging categories like ‘truck’ and ‘tractor’ inherently suffer from severe lack of texture and extremely small scales under the aerial bird’s-eye view, making them prone to false positives across all detectors, our method still maintains competitive or superior performance compared to the baselines. Specifically, MambaIR-YOLO reduces the misclassification rate between these hard examples and the background (Background FP/FN), further validating that the proposed lightweight state-space framework can effectively mitigate background interference and object detail degradation.

**Figure 9:**
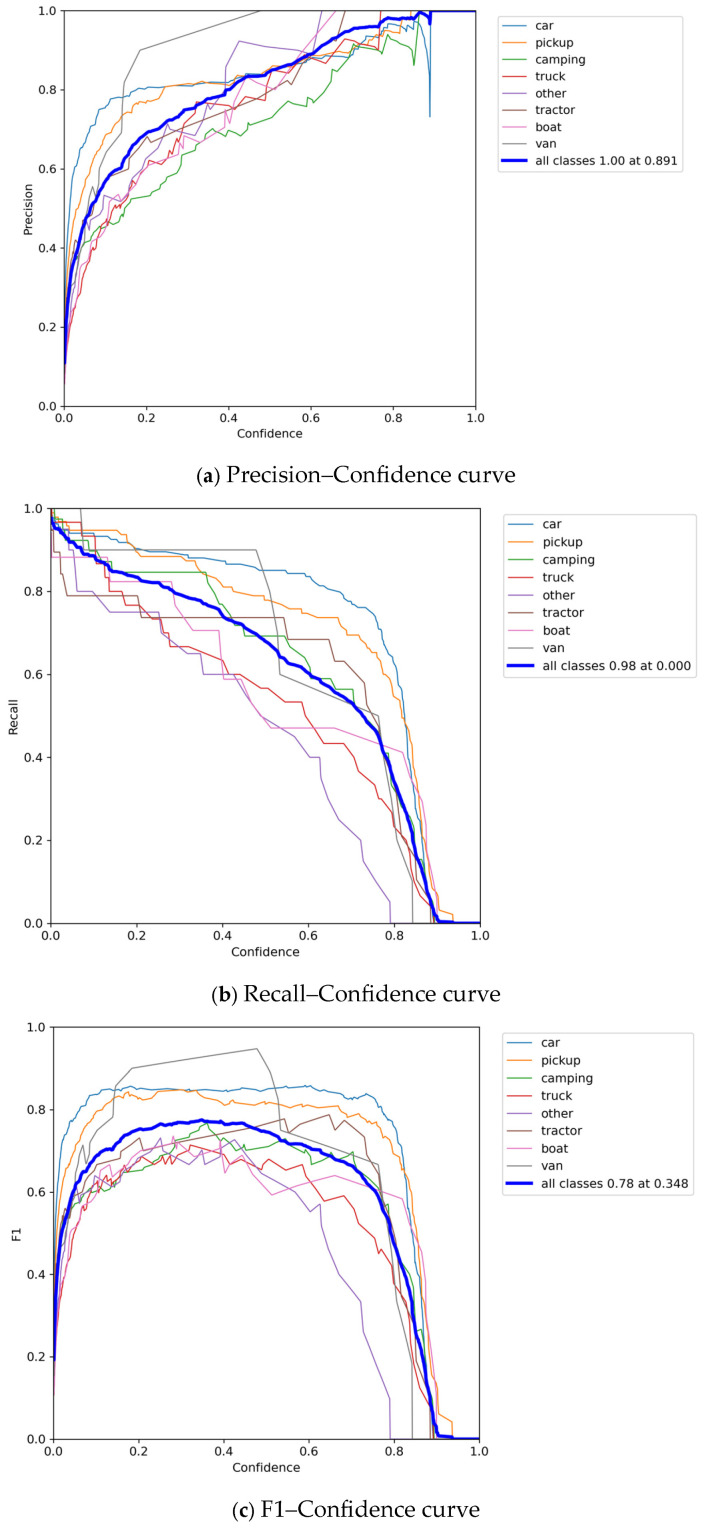

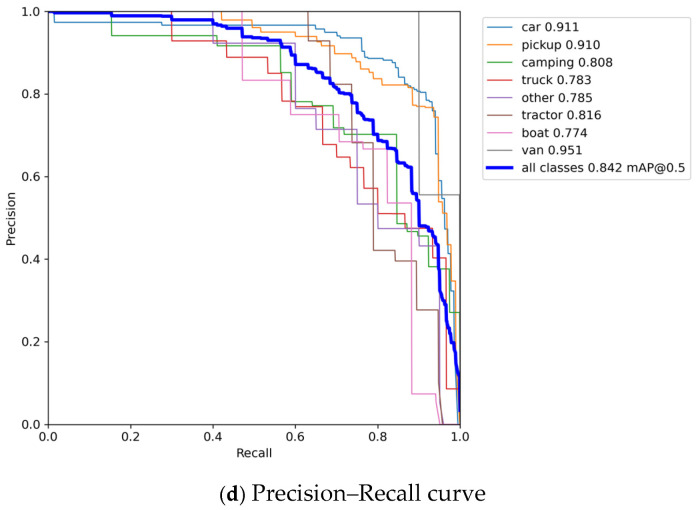
Evaluation curves for MambaIR-YOLO on the VEDAI dataset.

Figure 9 shows the evaluation curves for MambaIR-YOLO on the VEDAI dataset. Specifically, Figure 9a shows the Precision–Confidence curve, Figure 9b shows the Recall–Confidence curve, Figure 9c shows the F1–Confidence curve, and Figure 9d shows the Precision–Recall curve. As shown in Figure 9a, as the confidence threshold increases, low-confidence bounding boxes are gradually filtered out, and the model’s Precision shows an overall upward trend, indicating that a higher confidence threshold helps suppress false positives. As shown in Figure 9b, Recall gradually decreases as the confidence threshold increases, indicating that an excessively high threshold will cause some small-object bounding boxes to be filtered out, thereby increasing the risk of false negatives.

As shown in Figure 9c, the F1 score achieves optimal results at a confidence threshold of approximately 0.348, indicating that at this threshold, the model strikes a good balance between false positive suppression and target detection. For small-object detection tasks in aerial remote sensing, a confidence threshold that is too low can easily introduce false positives in complex backgrounds, while a threshold that is too high may lead to missed detections of weak or small objects; therefore, the F1–Confidence curve provides a reference for threshold selection in practical applications. The Precision–Recall curve shown in Figure 9d further indicates that MambaIR-YOLO exhibits relatively stable detection performance across most vehicle categories, with categories such as “car,” “pickup,” and “van” performing particularly well. However, for some categories, detection performance still has room for improvement due to factors such as sample size, target scale, and visual similarity.

Taken together, these visualization results show that MambaIR-YOLO not only achieves good detection performance in terms of quantitative metrics but also demonstrates more stable target localization capabilities in complex backgrounds, small-scale targets, dense targets, and low-contrast scenes. The confusion matrix and evaluation curves further illustrate that the method proposed in this paper delivers good overall performance in terms of multi-class recognition stability, false positive suppression, and small-target detection capability. The above results validate, from a visualization perspective, the effectiveness of the feature-level detail-guided, lightweight state-space modeling, and high-resolution single-scale detection strategies proposed in this paper for small-object detection in aerial remote sensing.

### 4.8. Generalization Capability Experiments and Analysis

To evaluate the cross-scenario generalization performance of MambaIR-YOLO, this paper transfers the network to the DroneVehicle [44], AI-TOD [45], and DOTA [1] datasets for training and testing, while maintaining consistency in the model’s key hyperparameters and training strategy. The experiments compared the standard YOLOv5s [12], the state-of-the-art multimodal network SuperYOLO [19], and the method proposed in this paper. mAP50 (%) was used as the quantitative evaluation metric, and the experimental results are shown in Table 5.

As shown in Table 5, first, on the DroneVehicle bimodal dataset, MambaIR-YOLO achieved an mAP50 of 81.85%, representing a 10.5% improvement over standard YOLOv5s and a 9.35% improvement over SuperYOLO, another lightweight multimodal network. This demonstrates that ODSSBlock and the FMB modulation module, driven by Lightweight Mamba, still possess robust local–global feature capabilities and superior modal fusion efficiency when handling large-scale, high-density, real-world aerial photography tasks.

Second, on the AI-TOD dataset, which specializes in extremely small targets, traditional detectors suffer from severely degraded shallow-layer high-frequency features due to continuous downsampling, limiting their accuracy. In contrast, MambaIR-YOLO significantly improves detection accuracy to 58.47 by leveraging high-resolution detail-guided constraints introduced during training via the feature-level MambaIR_SR branch, combined with a CIoU-NWD hybrid localization loss. This result supports the core advantage of our method in addressing the critical challenge of “feature degradation in extremely small objects.”

Finally, in the small-object category test on the DOTA dataset, although our model abandoned the conventional multi-scale prediction architecture in favor of a lightweight deployment—retaining only a high-resolution single-scale detection head—it still achieved an outstanding performance of 73.82%. This demonstrates that, through the top-down FPN path combined with the C3CA coordinate attention module, the high-resolution fused feature layer is capable of effectively concentrating key channels and precise spatial directional responses, ensuring accurate spatial localization of small-scale, densely clustered targets.

In summary, cross-dataset generalization experiments demonstrate that MambaIR-YOLO exhibits good robustness and practical deployment potential across various aerial photography and satellite remote sensing tasks, as well as under different modal inputs.

## 5. Conclusions

This paper addresses issues in small-object detection in aerial remote sensing—such as low object resolution, degradation of shallow-level details, strong interference from complex backgrounds, and difficulties in lightweight deployment—by proposing MambaIR-YOLO, a MambaIR-guided, lightweight, high-resolution, single-scale YOLO detection model. Based on the YOLOv5 framework, this model introduces improvements in three areas: feature-level detail guidance, lightweight state-space modeling, and high-resolution single-scale detection.

First, this paper constructs a feature-level MambaIR_SR training auxiliary branch. By utilizing shallow-layer detail features and deep-layer semantic features from the main network, it generates high-resolution detail guidance information, enabling the detection network to more thoroughly learn the edge, texture, and structural features of small objects during the training phase. This branch is removed during the inference phase; thus, it does not increase the number of parameters or computational load of the final deployed model. Second, we design the Lightweight Mamba-driven ODSSBlock and FMB. The ODSSBlock introduces lightweight state-space modeling capabilities into a C3-like dual-branch architecture to enhance the representation of local details and long-range context; the FMB is deployed in the shallow layers of the backbone, where it pre-modulates early high-frequency details via 3 × 3 depthwise convolutions and Lightweight Mamba, thereby mitigating the loss of detail in small objects during the downsampling process. Finally, we introduce the C3CA attention enhancement module at the Neck fusion node and adopt a high-resolution single-scale detection head, enabling the model to focus more on regions related to small objects and high-resolution localization information.

Experimental results on the VEDAI aerial remote sensing dataset show that MambaIR-YOLO achieves an mAP50 of 84.19 ± 0.03% with only 4.4588 M parameters and a computational cost of 19.97 GFLOPs. Compared with recent YOLO-series detectors and SuperYOLO, the proposed method achieves better overall detection accuracy while maintaining a lightweight model size and competitive inference speed, demonstrating a favorable balance among detection accuracy, model complexity, and deployment efficiency. Ablation experiments further validated the effectiveness of the MambaIR_SR, ODSSBlock, FMB, and C3CA modules, demonstrating that the improvement strategies proposed in this paper can mitigate the issues of detail degradation, insufficient context modeling, and complex background interference in small-object detection in aerial remote sensing from various perspectives.

Additionally, extensive generalization experiments on the DroneVehicle [41], AI-TOD [45], and DOTA [1] datasets demonstrated that the proposed model maintains excellent robustness and good generalization performance under various modal combinations, at extremely small scales, and in the presence of complex background interference.

Despite the advantages demonstrated in this study, the proposed MambaIR-YOLO framework has certain limitations. First, since our baseline architecture is built upon the YOLO series, the scope of current experimental comparisons is primarily focused on YOLO variants and a few representative lightweight networks. Comprehensive comparisons with other recently proposed specialized aerial detection non-YOLO frameworks or highly customized multi-scale fusion networks (e.g., UAV-YOLOv8, MFFSODNet) were restricted by the scope of this study and code availability. Second, while the ODSSBlock achieves a good balance between context modeling and parameter count, Mamba’s hardware-level optimization on low-power edge devices is not yet fully mature compared to highly optimized standard convolutions.

Therefore, our future work will focus on the following directions: (1) We plan to extend the proposed feature-level detail guidance and lightweight state-space modeling mechanisms to a broader range of state-of-the-art aerial detection architectures beyond the YOLO series to conduct more comprehensive comparative analyses. (2) We will explore lighter-weight state-space modeling structures and hardware-friendly operators to facilitate the seamless deployment of this high-resolution detection algorithm on resource-constrained UAV edge embedded devices, advancing the practical application of real-time aerial interpretation tasks.

## Figures and Tables

**Table 1:** Comparison results with state-of-the-art methods on the VEDAI dataset.

Method	Car	Pickup	Camping	Truck	Other	Tractor	Boat	Van	mAP50 (%)	mAP50–95(%)	Params (M)	GFLOPs (G)	FPS
YOLOv8n	75.20	72.10	69.40	72.00	61.50	39.20	49.40	18.00	57.10	33.70	3.00	8.10	239.56
YOLOv8s	74.60	63.50	73.90	59.60	64.00	71.40	71.60	50.60	66.10	38.80	11.12	28.50	**247.69**
YOLOv11n	79.90	71.70	72.60	56.20	52.20	60.50	64.80	58.30	64.50	37.60	2.58	6.30	197.24
YOLOv11s	85.30	68.60	68.40	73.80	63.20	63.00	66.30	66.50	69.40	40.20	9.41	21.30	173.05
YOLO26n	71.90	59.50	58.40	69.70	48.60	39.50	46.10	49.20	55.40	32.60	**2.37**	**5.20**	176.35
YOLO26s	82.70	71.00	78.70	**82.30**	50.90	55.80	60.80	57.50	67.50	39.70	9.46	20.50	126.90
SuperYOLO	**91.13**	85.66	79.30	70.18	57.33	80.41	60.24	76.50	75.09	43.72	4.84	17.98	218.12
Ours	**91.13**	**90.96**	**80.79**	78.26	**78.55**	**81.64**	**77.44**	**95.1**	**84.19**	**51.47**	4.45	19.97	196.36

Note: Bold values indicate the best performance.

**Table 2:** Comparison of the original YOLOv5s baseline and different ablation variants.

Model	MambaIR	ODSSBlock	FMB	C3CA	mAP50 (%)	mAP50–95 (%)
YOLOv5s	×	×	×	×	56.69	31.70
w/o MambaIR	×	√	√	√	80.00	48.80
w/o ODSSBlock	√	×	√	√	80.28	49.84
w/o FMB	√	√	×	√	77.73	48.73
w/o C3CA	√	√	√	×	76.15	45.27
Ours	√	√	√	√	**84.19**	**51.47**

Note: The symbol “√” indicates that the corresponding module is included, while “×” indicates that the module is not included. Bold values indicate the best performance.

**Table 3:** Comparison of model complexity and inference efficiency between the YOLOv5s baseline and different model variants.

Model	Params (M)	GFLOPs	FPS
YOLOv5s	7.041	**15.80**	212.77
w/o MambaIR	**4.458**	19.97	196.38
w/o ODSSBlock	5.870	22.53	**257.09**
w/o FMB	4.579	21.95	201.94
w/o C3CA	4.654	21.32	200.03
Ours	**4.458**	19.97	196.36

Note: Bold values indicate the best performance in terms of model complexity and inference efficiency among all compared methods.

**Table 4:** Statistics on model inference efficiency.

Metric	Value
Input mode	RGB + IR + MF
Input size	512 × 512
Batch size	1
Mean latency	5.093 ms/image
P95 latency	5.366 ms
FPS	196.36
Peak memory	371.81 MB

**Table 5:** Comparison of detection accuracy across different models on the generalization dataset.

Model	Params/M	DroneVehicle	AI-TOD	DOTA
YOLOv5s	7.71	71.35	41.20	63.15
SuperYOLO	4.85	72.5	52.16	68.73
Ours	**4.46**	**81.85**	**58.47**	**73.82**

Note: For monomodal datasets (AI-TOD and DOTA), the multimodal fusion front-end was removed, and models were evaluated using single RGB inputs. Bold values indicate the best performance.

**Figure A1:**
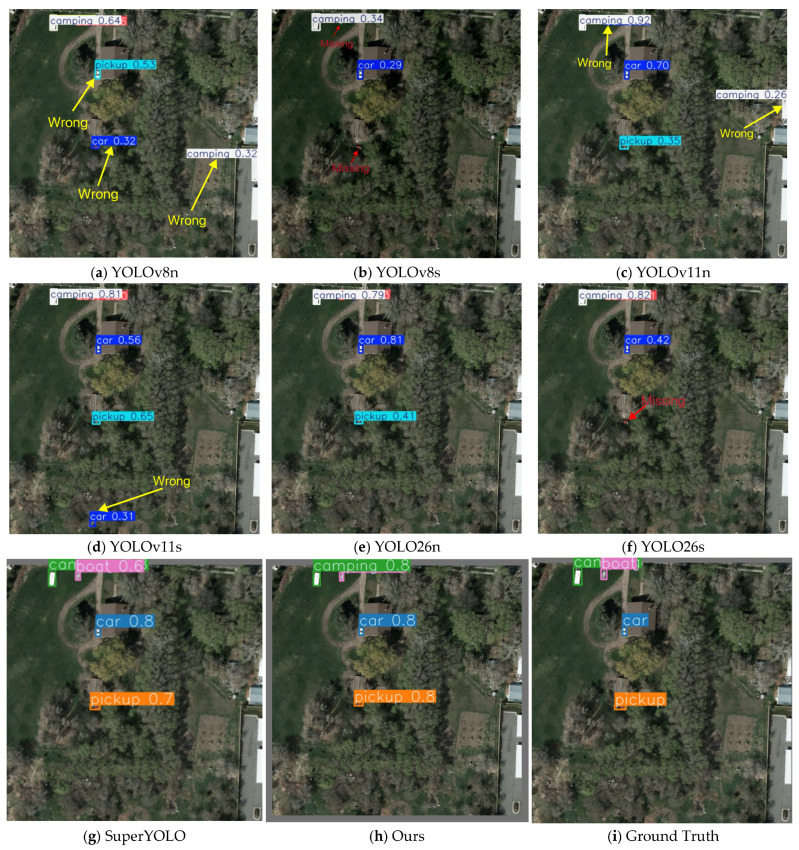
Additional qualitative comparison of detection results in challenging aerial scenes.

**Figure A2:**
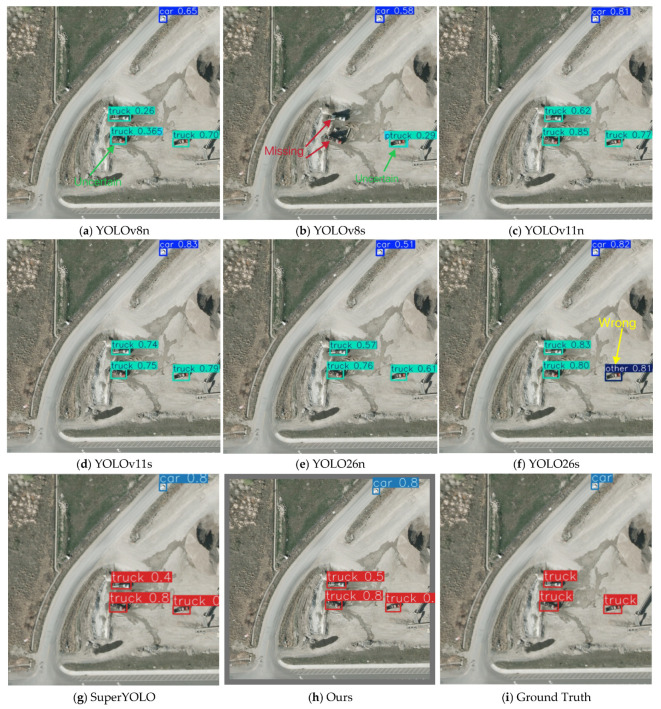
Additional qualitative comparison of detection results in challenging aerial scenes.

**Figure A3:**
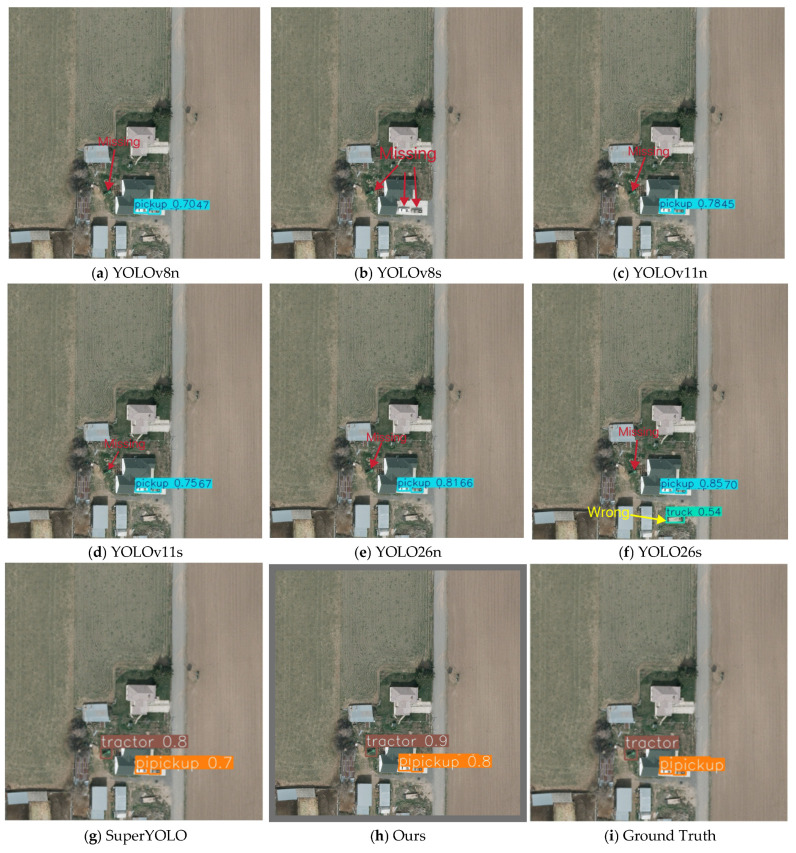
Additional qualitative comparison of detection results in challenging aerial scenes.

## Data Availability

The datasets used in this study are all publicly available. This manuscript encompasses all data that were produced or examined throughout the course of this study. Accompanying scripts and computational methods integral to the data’s creation will be made available in due course.

## Abbreviations

The following abbreviations are used in this manuscript:

AP	Average Precision
BCE	Binary Cross-Entropy
C3CA	C3 module with Coordinate and Channel Attention
CIoU	Complete Intersection over Union
IoU	Intersection over Union
CNN	Convolutional Neural Network
FMB	Feature Modulation Block
FPN	Feature Pyramid Network
FPS	Frames Per Second
GFLOPs	Giga Floating-Point Operations
IR	Infrared
RGB	Red, Green, and Blue
LADH	Lightweight Decoupled Head
MambaIR	Mamba for Image Restoration
MambaIR_SR	MambaIR Super-Resolution
mAP	mean Average Precision
mAP50	mean Average Precision at an IoU threshold of 0.50
NWD	Normalized Gaussian Wasserstein Distance
ODSSBlock	Object Detail State-Space Block
PAN	Path Aggregation Network
Params	Parameters
P95	95th Percentile Latency
SGD	Stochastic Gradient Descent
SR	Super-Resolution
UAV	Unmanned Aerial Vehicle

## Appendix A. Additional Qualitative Comparison Results

To provide a more comprehensive qualitative evaluation while maintaining the readability of Figure 7 in the main text, additional visual comparisons are presented in this Appendix A. These examples further compare the detection results of representative recent models, including YOLOv8, YOLO11, YOLO26, SuperYOLO, and the proposed MambaIR-YOLO, under challenging aerial scenes involving dense small objects, weak textures, complex backgrounds, and partial occlusion.
